# Supplementary material for: Transformer-based biomarker prediction from colorectal cancer histology: A large-scale multicentric study
Source: Cancer Cell. 2023 Sep 11;41(9):1650–1661.e4. doi: 10.1016/j.ccell.2023.08.002 (PMC10507381; doi:10.1016/j.ccell.2023.08.002)
Supplement: Document S1. Figures S1–S4 and Tables S1–S5 [file mmc1.pdf]

Cancer Cell, Volume *41*

Supplemental information

Transformer-based biomarker prediction from colorectal cancer histology: A large-scale multicentric study

Sophia J. Wagner, Daniel Reisenbüchler, Nicholas P. West, Jan Moritz Niehues, Jiefu Zhu, Sebastian Foersch, Gregory Patrick Veldhuizen, Philip Quirke, Heike I. Grabsch, Piet A. van den Brandt, Gordon G.A. Hutchins, Susan D. Richman, Tanwei Yuan, Rupert Langer, Josien C.A. Jenniskens, Kelly Offermans, Wolfram Mueller, Richard Gray, Stephen B. Gruber, Joel K. Greenson, Gad Rennert, Joseph D. Bonner, Daniel Schmolze, Jitendra Jonnagaddala, Nicholas J. Hawkins, Robyn L. Ward, Dion Morton, Matthew Seymour, Laura Magill, Marta Nowak, Jennifer Hay, Viktor H. Koelzer, David N. Church, TransSCOT consortium, Christian Matek, Carol Geppert, Chaolong Peng, Cheng Zhi, Xiaoming Ouyang, Jacqueline A. James, Maurice B. Loughrey, Manuel Salto-Tellez, Hermann Brenner, Michael Hoffmeister, Daniel Truhn, Julia A. Schnabel, Melanie Boxberg, Tingying Peng, and Jakob Nikolas Kather

Supplementary Figures

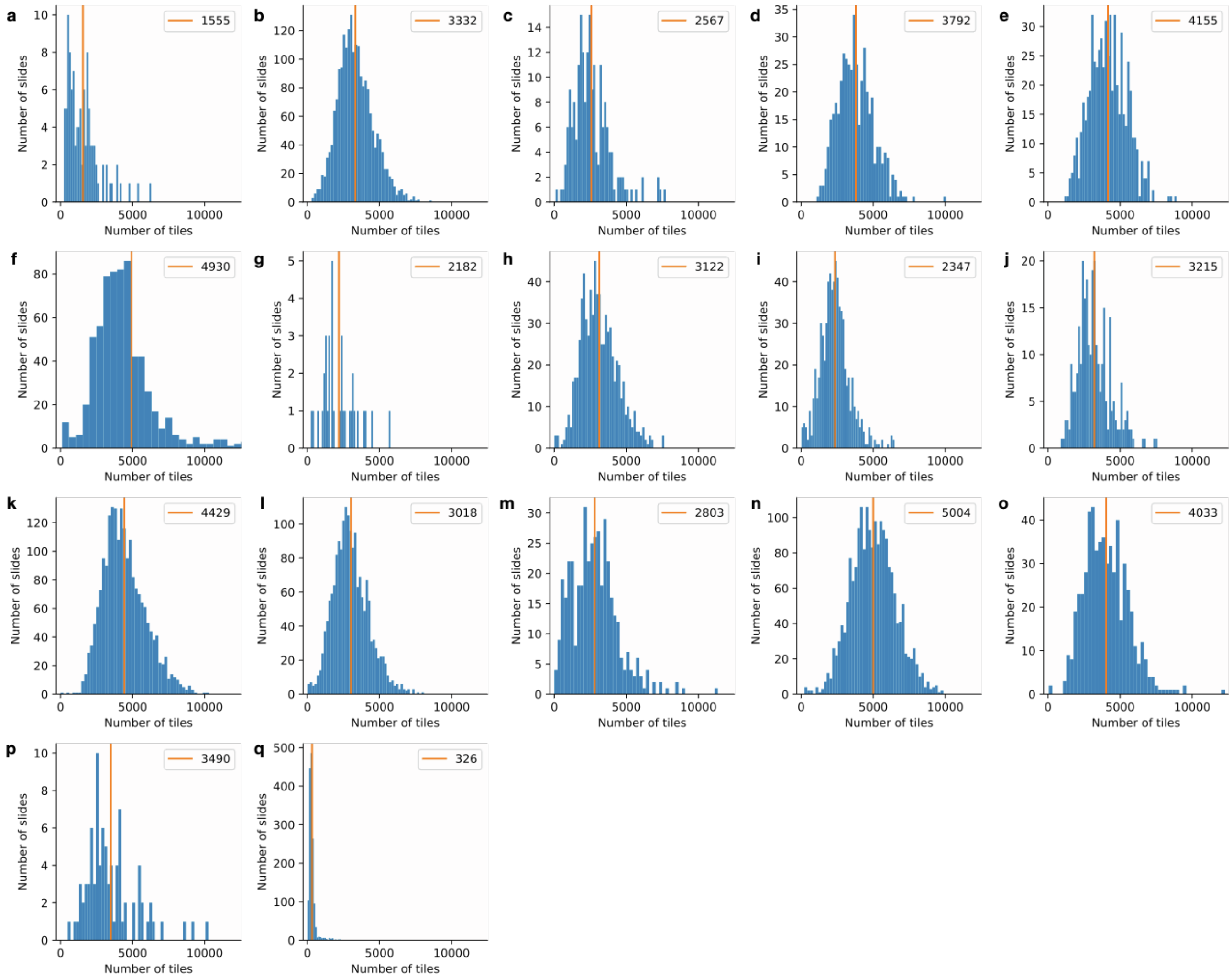

Supplementary Figure 1: **Distribution of the number of tiles per slide for all cohorts**, related to cohort overview in **Figure 1.** The mean of each distribution is highlighted in orange. a-o) Histogram of the distribution for all 15 cohorts of resections a) CPTAC, b) DACHS, c) DUSSEL, d) ERLANGEN, e) Epi700, f) FOXTROT, g) GUANGZHOU, h) MCO, i) MECC, j) MUNICH, k) NLCS, l) QUASAR, m) TCGA, n) TRANSCOT, and o) YCR-BCIP. p-q) Histogram of the distribution for the biopsy cohorts p) YCR-BCIP and q) MAINZ.

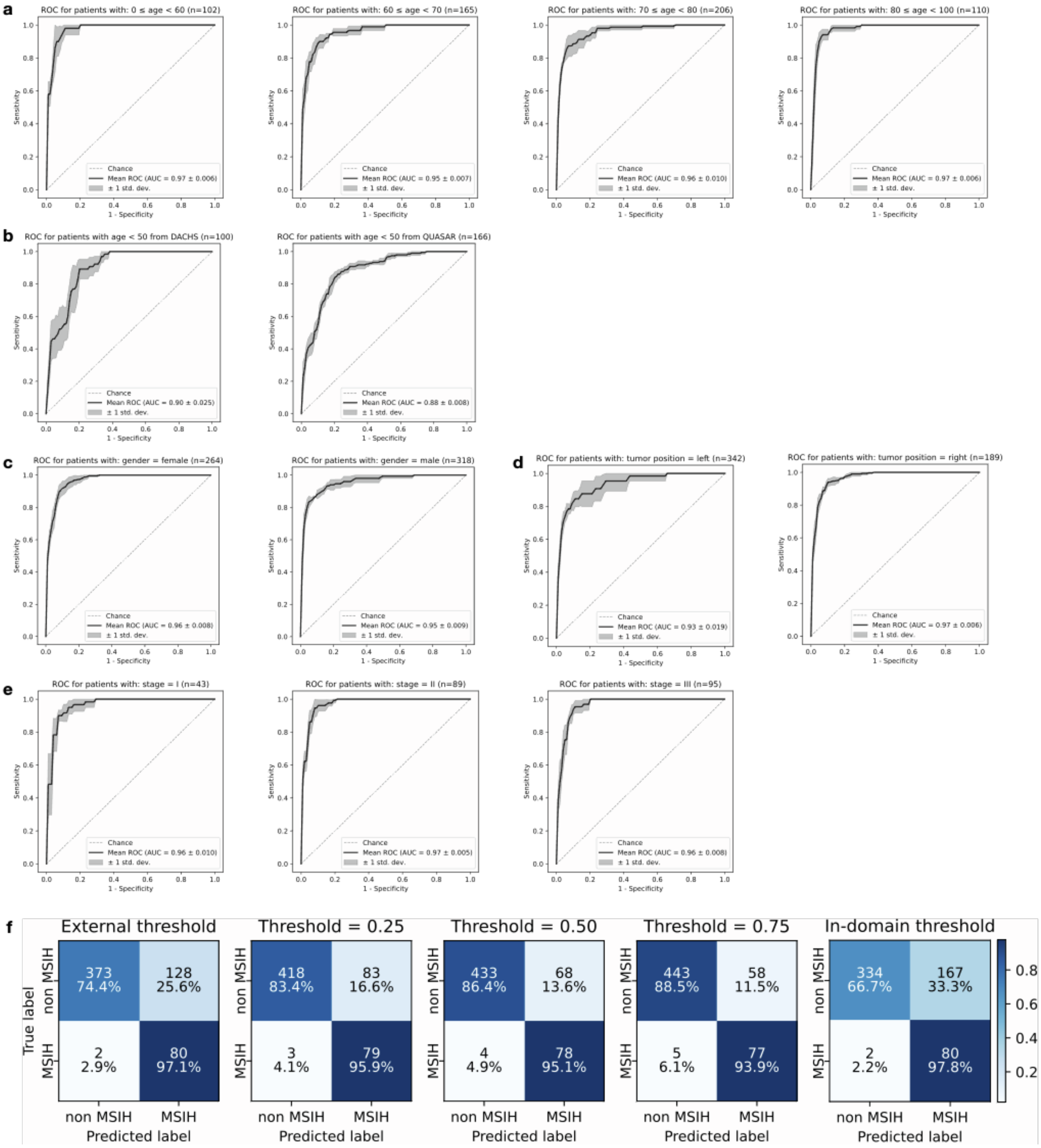

Supplementary Figure 2: **Analysis of clinico-pathological features with receiver operator curves (a-e) and confusion matrix for different classification thresholds (f)**, related to results in **Figure 2**. a) Age groups, below 60, between 60 and 70, between 70 and 80, and above 80 for multi-cohort model evaluated on YCR-BCIP. b) Early onset cancer: model trained on QUASAR, tested on DACHS with 100 patients younger than 50 years and model trained on DACHS, tested on QUASAR with 166 patients younger than 50 years. Both cohorts were chosen because they contain a sufficient number of patients under 50 in contrast to the other cohorts in this study. c-d) Multi-cohort model evaluated on YCR-BCIP. c) ROCs of female and male patients. d) ROCs for patiets with left- vs. right-sided tumors. d) ROCs for patients with tumors in stage I, II, and III. f) First column shows the threshold determined on the external tests, such that 0.95 sensitivity is reached. Second to forth column show fixed thresholds (0.25, 0.5, 0.75, respectively). Last column shows the threshold determined on the in-domain test set, such that 0.95 sensitivity is reached. The results show the average of the model trained on the large multi-centric cohort DACHS, NLCS, QUASAR, and TCGA across all five folds. Results of multi-cohort model evaluated on YCR-BCIP.

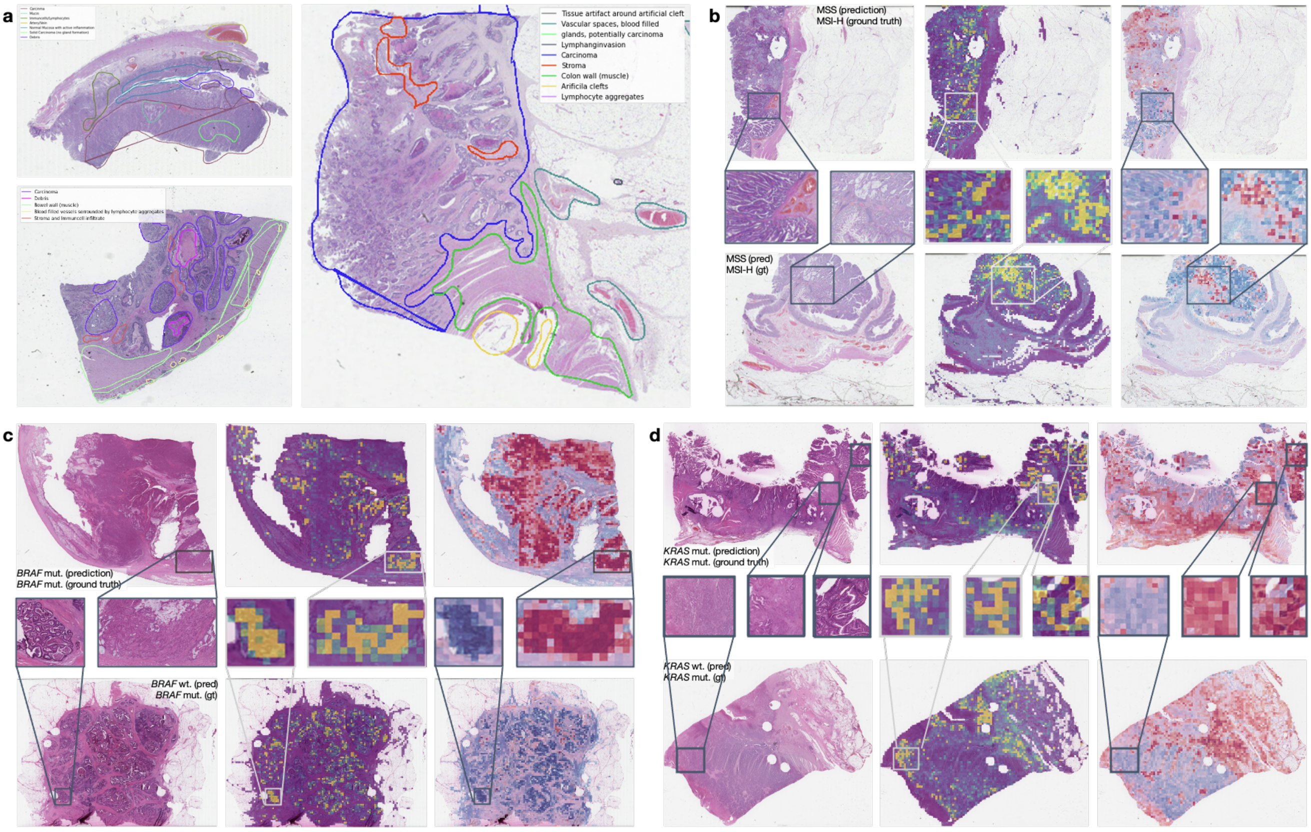

Supplementary Figure 3: **Annotations an additional cases for the interpretability analysis in Figure 3.** a) Manual annotation by a pathologist of the three test cases used for attention visualization from the YCR-BCIP cohort. b-d) Attention and classification score visualizations: left) original WSIs, center) attention score map, right) patch-wise classification score map. b) False negative cases in YCR-BCIP cohort for model trained on multi-cohort dataset. c) Model trained on the cohorts DACHS, QUASAR, MCO, NLCS, TCGA, for *BRAF* predictions, samples from the test cohort Epi700. d) Model trained on the cohorts DACHS, QUASAR, MCO, NLCS, TCGA, for *KRAS* predictions, samples from the test cohort Epi700.

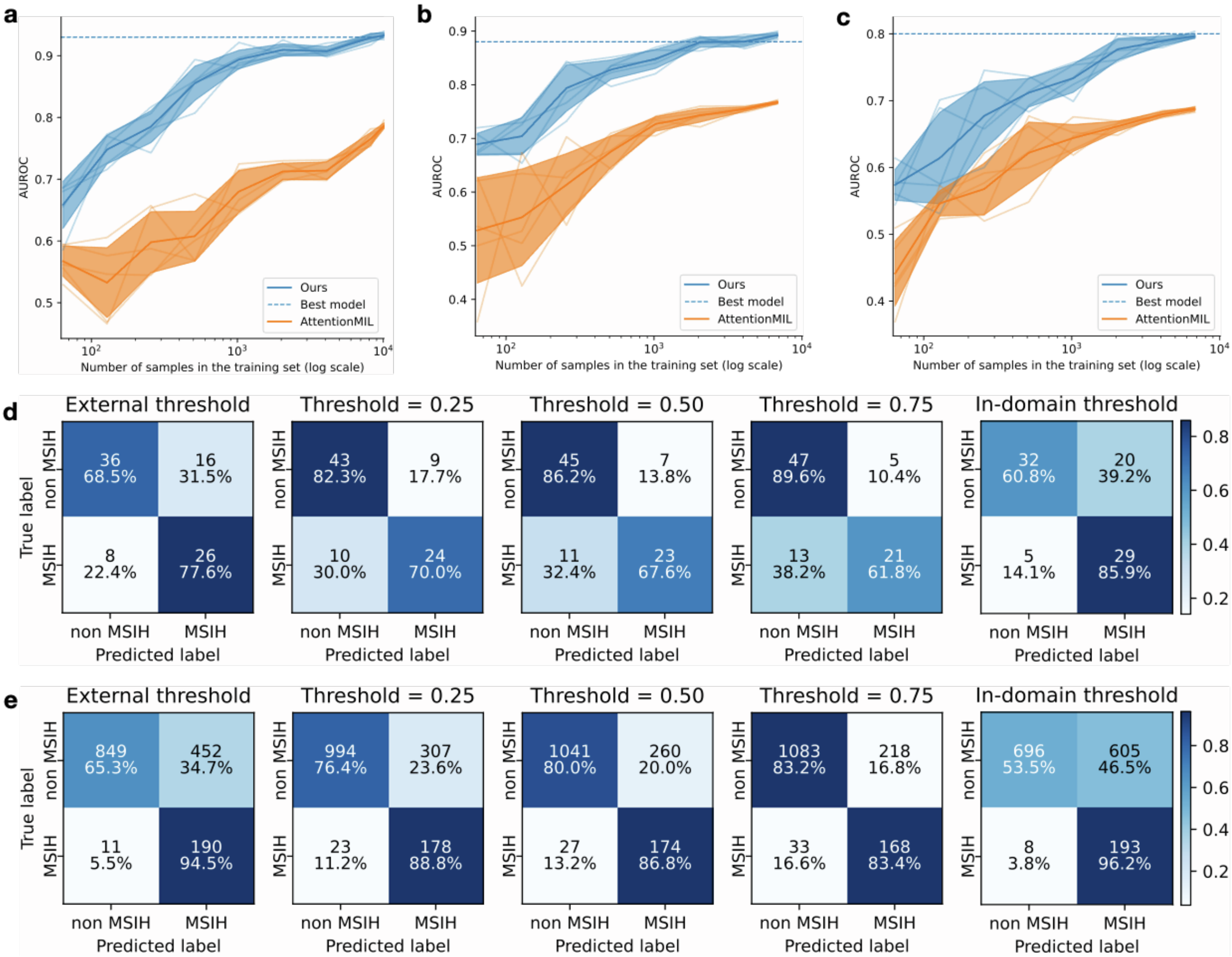

Supplementary Figure 4: Data efficiency analysis and confusion matrices, related to **Figure 4**. a-c) AUROC scores depending on the number of patients available for training. The samples were randomly drawn from all resection cohorts with available labels except the external test cohort. a) MSI prediction on YCR-BCIP. b) *BRAF* prediction on Epi700. c) *KRAS* prediction on Epi700. d-e) First column shows the threshold determined on the external tests, such that 0.95 sensitivity is reached. Second to forth column show fixed thresholds (0.25, 0.5, 0.75, respectively). Last column shows the threshold determined on the in-domain test set, such that 0.95 sensitivity is reached. d) Results of multi-cohort model evaluated on the biopsy cohort MAINZ. e) Results of multi-cohort model evaluated on the biopsy cohort YCR-BCIP.

Supplementary Tables

Suppl. Table 1: **Multi-cohort experiments with statistical endpoints,** related to **Figure 2.** Multi-cohort dataset consisting of CPTAC, DACHS, DUSSEL, Epi700, ERLANGEN, FOxTROT, MCO, MECC, MUNICH, QUASAR, RAINBOW, TCGA, TRANSCOT (all resection cohorts except YCR-BCIP and GUANGZHOU). The models were trained with HistAuGAN stain color augmentation, CTransPath as feature extractor and our transformer model with class token as aggregation model. The thresholds 0.9, 0.925, and 0.95 were determined on the in-domain test set and used for the evaluation on the external test sets. All results for sensitivity, negative predictive value (NPV), and sensitivity are averaged over the five folds.

**Train**	**Test**	**Target**	**AUROC mean**	**AUROC std dev**	**Sensitivity (0.95)**	**NPV (0.95)**	**Specificity (0.95)**	**Sensitivity (0.925)**	**NPV (0.925)**	**Specificity (0.925)**	**Sensitivity (0.9)**	**NPV (0.9)**	**Specificity (0.9)**
Multi-cohort dataset	Multi-cohort dataset	MSI high	0.93	0.0084	0.95	0.99	0.61	0.92	0.98	0.72	0.9	0.98	0.78
Multi-cohort dataset	YCR-BCIP-resections	MSI high	0.97	0.0041	0.995	0.998	0.41	0.99	0.995	0.56	0.98	0.995	0.67
Multi-cohort dataset	GUANGZHOU	MSI high	-	-	0.92	-	-	0.9	-	-	0.86	-	-
Multi-cohort dataset	YCR-BCIP-biopsies	MSI high	0.92	0.0066	0.99	0.995	0.31	0.98	0.99	0.44	0.96	0.99	0.54
Multi-cohort dataset	MAINZ	MSI high	0.86	0.0174	0.93	0.9	0.39	0.91	0.9	0.51	0.86	0.88	0.61
DACHS, QUASAR, NLCS, TCGA, MCO	DACHS, QUASAR, NLCS, TCGA, MCO	*BRAF*	0.88	0.0127	0.95	0.99	0.49	0.92	0.99	0.61	0.9	0.98	0.68
DACHS, QUASAR, NLCS, TCGA, MCO	Epi700	*BRAF*	0.88	0.0103	0.94	0.98	0.55	0.91	0.98	0.65	0.88	0.97	0.71
DACHS, QUASAR, NLCS, TCGA, MCO	DACHS, QUASAR, NLCS, TCGA, MCO	*KRAS*	0.71	0.0053	0.95	0.87	0.18	0.93	0.93	0.23	0.9	0.84	0.29
DACHS, QUASAR, NLCS, TCGA, MCO	Epi700	*KRAS*	0.80	0.0124	0.98	0.95	0.16	0.98	0.93	0.21	0.97	0.93	0.28

Suppl. Table 2: Ablation study on architecture choices, related to **Figure 2**. The models were trained with the same pre-processing and feature extractor, only varying the architecture of the aggregation model. All models were trained with 5-fold cross validation.

**Number**	**Train**	**Test**	**Target**	**Normalization**	**Feature Extraction**	**Aggregation Model**	**AUROC mean**	**AUROC std dev**	**AUPRC mean**	**AUPRC std dev**	**F1 (0.5) mean**	**F1 (0.5) std dev**	**F1 (gmean) mean**	**F1 (gmean) std dev**
2.1.1	DACHS, NLCS, QUASAR, TCGA	DACHS, NLCS, QUASAR, TCGA	MSI-H	Macenko	CTransPath[29]	Transformer with class token (ours)	0.95	0.0078	0.74	0.0284	0.83	0.1257	0.80	0.1370
2.2.1	DACHS, NLCS, QUASAR, TCGA	YCR-BCIP	MSI-H	Macenko	CTransPath[29]	Transformer with class token (ours)	**0.97**	0.0041	0.83	0.0266	**0.83**	0.1145	**0.84**	0.1108
2.3.1	DACHS, NLCS, QUASAR, TCGA	YCR-BCIP-biopsies	MSI-H	Macenko	CTransPath[29]	Transformer with class token (ours)	**0.91**	0.0094	0.63	0.0149	**0.77**	0.1616	**0.74**	0.1622
2.1.2	DACHS, NLCS, QUASAR, TCGA	DACHS, NLCS, QUASAR, TCGA	MSI-H	Macenko	CTransPath[29]	Transformer with global averaging (ours)	0.95	0.0091	0.76	0.0224	0.83	0.1268	0.81	0.1322
2.2.2	DACHS, NLCS, QUASAR, TCGA	YCR-BCIP	MSI-H	Macenko	CTransPath[29]	Transformer with global averaging (ours)	**0.97**	0.0042	**0.84**	0.0131	0.82	0.1272	0.83	0.1191
2.3.2	DACHS, NLCS, QUASAR, TCGA	YCR-BCIP-biopsies	MSI-H	Macenko	CTransPath[29]	Transformer with global averaging (ours)	**0.91**	0.0078	**0.64**	0.0206	0.75	0.1795	**0.74**	0.1582
2.1.3	DACHS, NLCS, QUASAR, TCGA	DACHS, NLCS, QUASAR, TCGA	MSI-H	Macenko	CTransPath[29]	AttentionMIL[23]	0.94	0.0103	0.71	0.0184	0.79	0.1477	0.77	0.1543
2.2.3	DACHS, NLCS, QUASAR, TCGA	YCR-BCIP	MSI-H	Macenko	CTransPath[29]	AttentionMIL[23]	0.96	0.0025	0.80	0.0101	0.78	0.1413	0.82	0.1202
2.3.3	DACHS, NLCS, QUASAR, TCGA	YCR-BCIP-biopsies	MSI-H	Macenko	CTransPath[29]	AttentionMIL[23]	0.90	0.0042	0.60	0.0154	0.76	0.1601	**0.74**	0.1595
2.1.4	DACHS, NLCS, QUASAR, TCGA	DACHS, NLCS, QUASAR, TCGA	MSI-H	Macenko	CTransPath[29]	TransMIL[32]	0.94	0.0101	0.72	0.0379	0.82	0.1387	0.79	0.1500
2.2.4	DACHS, NLCS, QUASAR, TCGA	YCR-BCIP	MSI-H	Macenko	CTransPath[29]	TransMIL[32]	0.96	0.0033	0.79	0.0200	0.84	0.1157	0.83	0.1155
2.3.4	DACHS, NLCS, QUASAR, TCGA	YCR-BCIP-biopsies	MSI-H	Macenko	CTransPath[29]	TransMIL[32]	0.89	0.0122	0.57	0.0178	0.72	0.2215	0.70	0.1736

Suppl. Table 3: STARD (STAndards for the Reporting of Diagnostic accuracy studies) Checklist, related to STAR Methods**.**

**Section & Topic**	**No**	**Item**	**Reported**
TITLE OR ABSTRACT	1	Identification as a study of diagnostic accuracy using at least one measure of accuracy (such as sensitivity, specificity, predictive values, or AUC)	yes
ABSTRACT	2	Structured summary of study design, methods, results, and conclusions (for specific guidance, see STARD for Abstracts)	yes
INTRODUCTION	3	Scientific and clinical background, including the intended use and clinical role of the index test	yes
	4	Study objectives and hypotheses	yes
METHODS<br>Study design	5	Whether data collection was planned before the index test and reference standard were performed (prospective study) or after (retrospective study)	
METHODS<br>Participants	6	Eligibility criteria	
	7	On what basis potentially eligible participants were identified (such as symptoms, results from previous tests, inclusion in registry)	
	8	Where and when potentially eligible participants were identified (setting, location and dates)	
	9	Whether participants formed a consecutive, random or convenience series	
METHODS<br>Test methods	10a	Index test, in sufficient detail to allow replication	yes
	10b	Reference standard, in sufficient detail to allow replication	yes
	11	Rationale for choosing the reference standard (if alternatives exist)	
	12a	Definition of and rationale for test positivity cut-offs or result categories of the index test, distinguishing pre-specified from exploratory	yes
	12b	Definition of and rationale for test positivity cut-offs or result categories of the reference standard, distinguishing pre-specified from exploratory	
	13a	Whether clinical information and reference standard results were available to the performers/readers of the index test	
	13b	Whether clinical information and index test results were available to the assessors of the reference standard	
METHODS<br>Analysis	14	Methods for estimating or comparing measures of diagnostic accuracy	yes
	15	How indeterminate index test or reference standard results were handled	
	16	How missing data on the index test and reference standard were handled	yes
	17	Any analyses of variability in diagnostic accuracy, distinguishing pre-specified from exploratory	
	18	Intended sample size and how it was determined	yes
RESULTS<br>Participants	19	Flow of participants, using a diagram	

Section & Topic	No	Item	Reported
	20	Baseline demographic and clinical characteristics of participants	yes
	21a	Distribution of severity of disease in those with the target condition	
	21b	Distribution of alternative diagnoses in those without the target condition	yes
	22	Time interval and any clinical interventions between index test and reference standard	
RESULTS<br>Test results	23	Cross tabulation of the index test results (or their distribution) by the results of the reference standard	
	24	Estimates of diagnostic accuracy and their precision (such as 95% confidence intervals)	yes
	25	Any adverse events from performing the index test or the reference standard	
DISCUSSION	26	Study limitations, including sources of potential bias, statistical uncertainty, and generalisability	yes
	27	Implications for practice, including the intended use and clinical role of the index test	
OTHER<br>INFORMATION	28	Registration number and name of registry	
	29	Where the full study protocol can be accessed	
	30	Sources of funding and other support; role of funders	yes

Suppl. Table 4: Patient cohorts used in this study and their characteristics, related to Figure 1. Clinico-pathological data were provided by the respective study principal investigators. In all cases, the TNM version from the original study registry was used. Information about the localization of the tumor was either provided as a binary variable (left-sided vs. right-sided) by the study site or assigned by the authors as follows: the cecum, ascending colon, hepatic flexure and transverse colon were defined as a right-sided tumor location whereas the splenic flexure, descending colon, sigmoid colon and rectum were defined as left-sided. *Number of patients before dropout of samples. **for the MECC cohort, these statistics refer to the cases with available MSI/dMMR status only.

	**CPTAC**	**DACHS**	**DUSSEL**	**Epi700**	**ERLANGEN**	**FOxTROT**	**GUANGZHOU**	**MAINZ**	**MCO**	**MECC****	**MUNICH**	**NLCS**	**QUASAR**	**TCGA**	**TRANSCOT**	**YCR-BCIP**	**YCR-BCIP biopsies**
**Origin**	United States	Germany	Germany	Northern Ireland	Germany	United Kingdom	China	German y	Australia	Israel	Germany	Nether-lands	United Kingdom	United States	United Kingdom	United Kingdom	United Kingdom
**Number of patients***	110	2448	330	661	627	1053	35	90	1511	683	292	2452	2190	632	1988	889	1557
**WSI format**	SVS	SVS	SVS	SVS	MRXS	SVS	MRXS	SVS	SVS	TIF	SVS	TIFF/SVS	SVS	SVS	SVS	SVS	SVS
**MSI-H/dMMR ground truth**	MuTect2[38]	PCR 3-plex	IHC 2-plex	PCR/IHC consensus	IHC 4-plex	IHC 4-plex	IHC 4-plex	IHC 4-plex	IHC 4-plex	PCR 5-plex	IHC 4-plex	IHC 2-plex	IHC 4-plex / IHC 2-plex	PCR 5-plex[72]	IHC	IHC 4-plex	IHC 4-plex
**MSI-H/dMMR, n (%)**	24 (22%)	210 (9%)	45 (14%)	134 (20%)	113 (18%)	185 (18%)	35 (100%)	36 (40%)	238 (16%)	106 (16%)	34 (12%)	259 (11%)	246 (11%)	65 (10%)	229 (12%)	129 (15%)	211 (14%)
**MSS/pMMR, n (%)**	81 (74%)	1836 (75%)	268 (81%)	469 (71%)	407 (65%)	728 (69%)	0 (0%)	54 (60%)	1268 (85%)	577 (84%)	258 (88%)	2193 (89%)	1529 (70%)	392 (62%)	1759 (88%)	760 (85%)	1346 (86%)
**Mean age at diagnosis (std. dev.)**	65.67 (11.38)	68.46 (10.82)	68.57 (11.77)	70.63 (11.4)	N/A	N/A	N/A	N/A	68.4 (12.51)	69.8	56.1 (11.84)	73.71 (6.04)	62.20 (9.60)	66.42 (12.67)	63.84 (9.11)	70.31 (9.97)	71.79 (9.97)
**Colon cancer, n (%)**	110 (100%)	1488 (61%)	204 (62%)	659 (99.7%)	N/A	N/A	N/A	N/A	955 (63%)	530 (78%)	N/A	1730 (71%)	1474 (67%)	341 (54%)	N/A	667 (75%)	876 (56%)
**Rectal cancer, n (%)**	0 (0%)	960 (39%)	116 (35%)	2 (0.3%)	N/A	N/A	N/A	N/A	552 (37%)	123 (18%)	N/A	722 (29%)	526 (24%)	118 (19%)	N/A	215 (24%)	662 (43%)
**Site unknown, n (%)**	0 (0%)	0 (0%)	10 (3%)	0 (0%)	N/A	N/A	N/A	N/A	4 (0%)	30 (4%)	N/A	0 (0%)	190 (9%)	173 (27%)	N/A	7 (1%)	19 (1%)
**Female, n (%)**	65 (59%)	1012 (41%)	181 (55%)	303 (46%)	N/A	N/A	N/A	N/A	685 (45%)	320 (47%)	132 (45%)	1079 (44%)	848 (39%)	292 (46%)	802 (40%)	395 (44%)	620 (40%)
**Male, n (%)**	45 (41%)	1436 (59%)	149 (45%)	358 (54%)	N/A	N/A	N/A	N/A	826 (55%)	363 (53%)	160 (55%)	1373 (56%)	1334 (61%)	322 (51%)	1186 (60%)	494 (56%)	933 (60%)
**gender unknown**	0 (0%)	0 (0%)	0 (0%)	0 (0%)	N/A	N/A	N/A	N/A	0 (0%)	0 (0%)	0 (0%)	0 (0%)	8 (0%)	18 (3%)	0 (0%)	0 (0%)	4 (0%)
**UICC stage I, n (%)**	12 (11%)	485 (20%)	76 (23%)	0 (0%)	N/A	N/A	N/A	N/A	289 (19%)	94 (14%)	52 (18%)	485 (20%)	1 (0%)	76 (12%)	N/A	169 (19%)	2 (0%)
**UICC stage II, n (%)**	42 (38%)	801 (33%)	138 (42%)	394 (60%)	N/A	N/A	N/A	N/A	542 (36%)	335 (49%)	118 (40%)	918 (37%)	1988 (91%)	166 (26%)	N/A	317 (36%)	2 (0%)
**UICC stage III, n (%)**	48 (44%)	822 (34%)	110 (33%)	267 (40%)	N/A	N/A	N/A	N/A	503 (33%)	123 (18%)	82 (28%)	641 (26%)	192 (9%)	140 (22%)	N/A	370 (42%)	5 (0%)
**UICC stage IV, n**	8	337	6	0	N/A	N/A	N/A	N/A	177	67	39	341	0	63	N/A	0	0

	CPTAC	DACHS	DUSSEL	Epi700	ERLANGEN	FOxTROT	GUANGZHOU	MAINZ	MCO	MECC**	MUNICH	NLCS	QUASAR	TCGA	TRANSCOT	YCR-BCIP	YCR-BCIP biopsies
**(%)**	(7%)	(14%)	(2%)	(0%)					(12%)	(10%)	(13%)	(14%)	(0%)	(10%)		(0%)	(0%)
**UICC stage unknown, n (%)**	0 (0%)	3 (0%)	0 (0%)	0 (0%)	N/A	N/A	N/A	N/A	0 (0%)	64 (9%)	1 (0%)	67 (3%)	9 (0%)	187 (30%)	N/A	33 (3%)	1548 (99%)
***BRAF* mutation, n (%)**	N/A	151 (6%)	N/A	91 (14%)	N/A	N/A	N/A	N/A	190 (13%)	49 (7%)	N/A	305 (12%)	120 (5%)	63 (10%)	N/A	75 (8%)	139 (9%)
***BRAF* wild type, n (%)**	N/A	1930 (79%)	N/A	550 (84%)	N/A	N/A	N/A	N/A	1271 (84%)	570 (83%)	N/A	1733 (71%)	1358 (62%)	471 (75%)	N/A	32 (4%)	36 (2%)
***BRAF* status unknown, n (%)**	N/A	367 (15%)	N/A	16 (2%)	N/A	N/A	N/A	N/A	50 (3%)	64 (9%)	N/A	414 (17%)	712 (33%)	98 (15%)	N/A	782 (88%)	1382 (89%)
***KRAS* mutation, n (%)**	N/A	677 (28%)	N/A	247 (38%)	N/A	N/A	N/A	N/A	460 (30%)	252 (37%)	N/A	698 (28%)	555 (25%)	218 (34%)	N/A	N/A	N/A
***KRAS* wild type, n (%)**	N/A	1397 (57%)	N/A	398 (61%)	N/A	N/A	N/A	N/A	1001 (66%)	405 (59%)	N/A	1335 (54%)	882 (40%)	316 (50%)	N/A	N/A	N/A
***KRAS* status unknown**	N/A	347 (15%)	N/A	12 (2%)	N/A	N/A	N/A	N/A	50 (3%)	26 (4%)	N/A	419 (17%)	753 (35%)	98 (16%)	N/A	N/A	N/A
**right-sided tumor, n (%)**	58 (53%)	819 (33%)	72 (22%)	375 (57%)	N/A	N/A	N/A	N/A	589 (39%)	238 (35%)	53 (18%)	946 (39%)	754 (34%)	176 (28%)	779 (39%)	331 (37%)	395 (25%)
**left-sided tumor, n (%)**	51 (46%)	1607 (66%)	226 (68%)	280 (42%)	N/A	N/A	N/A	N/A	918 (61%)	409 (60%)	239 (82%)	1506 (61%)	1158 (53%)	248 (39%)	1180 (60%)	486 (55%)	1055 (68%)
**sidedness unknown, n (%)**	1 (1%)	22 (1%)	32 (10%)	6 (1%)	N/A	N/A	N/A	N/A	4 (0%)	36 (5%)	0 (0%)	0 (0%)	150 (13%)	208 (33%)	29 (1%)	72 (8%)	107 (7%)

Suppl. Table 5 Mean AUROC scores of 5-fold CV training on single cohorts, evaluated on all other cohorts, related to **Figure 2**. The training cohorts are listed in the columns and entries in the diagonal are in-domain test results.

Train (↓)	NLCS	DACHS	TRANSCOT	QUASAR	MCO	YCR-BCIP	FOxTROT	MECC	Epi700	ERLANGEN	TCGA	MUNICH	DUSSEL	CPTAC
**NLCS**	0.93	0.92	0.93	0.93	0.93	0.95	0.88	0.78	0.92	0.72	0.89	0.85	0.83	0.89
**DACHS**	0.91	0.96	0.91	0.92	0.93	0.92	0.86	0.76	0.91	0.74	0.87	0.83	0.83	0.87
**TRANSCOT**	0.91	0.91	0.93	0.93	0.92	0.95	0.87	0.76	0.93	0.75	0.89	0.86	0.80	0.85
**QUASAR**	0.93	0.92	0.93	0.96	0.93	0.95	0.89	0.76	0.93	0.72	0.88	0.88	0.81	0.91
**MCO**	0.92	0.91	0.92	0.93	0.94	0.94	0.88	0.78	0.92	0.75	0.87	0.87	0.82	0.88
**YCR-BCIP**	0.90	0.87	0.91	0.91	0.92	0.95	0.86	0.76	0.91	0.76	0.87	0.86	0.82	0.92
**FOxTROT**	0.83	0.82	0.89	0.82	0.80	0.87	0.81	0.70	0.84	0.71	0.80	0.77	0.79	0.82
**MECC**	0.85	0.82	0.88	0.86	0.85	0.88	0.81	0.77	0.86	0.68	0.81	0.77	0.80	0.79
**Epi700**	0.90	0.90	0.92	0.93	0.93	0.94	0.87	0.77	0.95	0.72	0.87	0.88	0.80	0.88
**ERLANGEN**	0.80	0.81	0.86	0.78	0.80	0.85	0.77	0.66	0.82	0.76	0.76	0.80	0.77	0.68
**TCGA**	0.82	0.86	0.84	0.85	0.83	0.88	0.80	0.72	0.85	0.70	0.83	0.84	0.76	0.82
**MUNICH**	0.77	0.78	0.79	0.80	0.80	0.84	0.74	0.65	0.81	0.72	0.76	0.85	0.76	0.71
**DUSSEL**	0.72	0.75	0.83	0.75	0.71	0.81	0.74	0.65	0.74	0.70	0.69	0.67	0.71	0.69
**CPTAC**	0.74	0.74	0.78	0.74	0.71	0.81	0.72	0.67	0.77	0.70	0.69	0.67	0.71	0.73
