# Supplementary material for: Transformer-based biomarker prediction from colorectal cancer histology: A large-scale multicentric study
Source: Cancer Cell. 2023 Sep 11;41(9):1650–1661.e4. doi: 10.1016/j.ccell.2023.08.002 (PMC10507381; doi:10.1016/j.ccell.2023.08.002)
Supplement: Document S2. Article plus supplemental information [file mmc2.pdf]

Cancer Cell

Article

Transformer-based biomarker prediction from colorectal cancer histology: A large-scale multicentric study

Graphical abstract

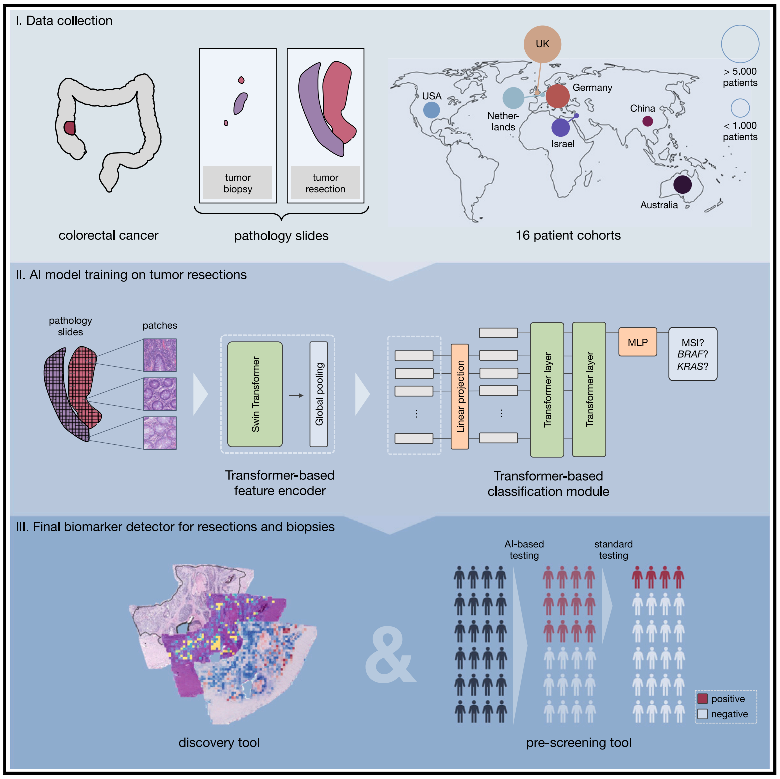

Highlights

- AI-based prediction of biomarkers (MSI, *BRAF*, and *KRAS*) using transformers
- MSI prediction reaches clinical-grade performance on biopsies of colorectal cancer
- Transformer-based biomarker prediction generalizes better and is more data efficient
- Large-scale multi-cohort evaluation on over 13,000 patients from 16 cohorts

Authors

Sophia J. Wagner, Daniel Reisenbüchler, Nicholas P. West, ..., Melanie Boxberg, Tingying Peng, Jakob Nikolas Kather

Correspondence

tingying.peng@helmholtz-munich.de (T.P.), jakob_nikolas.kather@tu-dresden.de (J.N.K.)

In brief

Wagner et al. show that transformer-based prediction of biomarkers from histology substantially improves the performance, generalizability, data efficiency, and interpretability as compared with current state-of-the-art algorithms. The method significantly outperforms existing approaches for microsatellite instability detection in surgical resections and reaches clinical-grade performance on biopsies of colorectal cancer, solving a long-standing diagnostic problem.

Check for updates

Wagner et al., 2023, Cancer Cell *41*, 1650–1661
September 11, 2023 © 2023 The Author(s). Published by Elsevier Inc.
https://doi.org/10.1016/j.ccell.2023.08.002

CellPress

CellPress
OPEN ACCESS

Cancer Cell

Article

Transformer-based biomarker prediction from colorectal cancer histology: A large-scale multicentric study

Sophia J. Wagner,[1,2,3] Daniel Reisenbüchler,[1] Nicholas P. West,[4] Jan Moritz Niehues,[3] Jiefu Zhu,[3] Sebastian Foersch,[4] Gregory Patrick Veldhuizen,[3] Philip Quirke,[5] Heike I. Grabsch,[5,6] Piet A. van den Brandt,[7] Gordon G.A. Hutchins,[5] Susan D. Richman,[5] Tanwei Yuan,[8] Rupert Langer,[9] Josien C.A. Jenniskens,[7] Kelly Offermans,[7] Wolfram Mueller,[10] Richard Gray,[11] Stephen B. Gruber,[12] Joel K. Greenson,[13] Gad Rennert,[14,15] Joseph D. Bonner,[14] Daniel Schmolze,[12] Jitendra Jonnagaddala,[16] Nicholas J. Hawkins,[17] Robyn L. Ward,[17,18] Dion Morton,[19]

(Author list continued on next page)

[1]Helmholtz Munich – German Research Center for Environment and Health, Munich, Germany
[2]School of Computation, Information and Technology, Technical University of Munich, Munich, Germany
[3]Else Kroener Fresenius Center for Digital Health (EFFZ), Technical University Dresden, Dresden, Germany
[4]Institute of Pathology, University Medical Center Mainz, Mainz, Germany
[5]Division of Pathology and Data Analytics, Leeds Institute of Medical Research at St James's, University of Leeds, Leeds, UK
[6]Department of Pathology, GROW School for Oncology and Developmental Biology, Maastricht University Medical Center+, Maastricht, the Netherlands
[7]Department of Epidemiology, Maastricht University Medical Center+, Maastricht, the Netherlands
[8]Division of Clinical Epidemiology and Aging Research, German Cancer Research Center (DKFZ), Heidelberg, Germany
[9]Institute of Pathology und Molecular Pathology, Johannes Kepler University Hospital Linz, Linz, Österreich
[10]Gemeinschaftspraxis Pathologie, Starnberg, Germany
[11]Nuffield Department of Population Health, University of Oxford, Oxford, UK
[12]Center for Precision Medicine and Department of Medical Oncology, City of Hope National Medical Center, Duarte, CA, USA
[13]Department of Pathology, City of Hope Comprehensive Cancer Center, Duarte, CA, USA
[14]Department of Community Medicine & Epidemiology, Lady Davis Carmel Medical Center, Ruth & Bruce Rappaport Faculty of Medicine, Technion-Israel Institute of Technology, Haifa, Israel
[15]Steve and Cindy Rasmussen Institute for Genomic Medicine, Lady Davis Carmel Medical Center and Technion Faculty of Medicine, Clalit National Cancer Control Center, Haifa, Israel
[16]School of Population Health, Faculty of Medicine and Health, UNSW Sydney, Sydney, NSW, Australia
[17]School of Medical Sciences, Faculty of Medicine and Health, UNSW Sydney, Sydney, NSW, Australia
[18]Faculty of Medicine and Health, The University of Sydney, Sydney, NSW, Australia

(Affiliations continued on next page)

SUMMARY

Deep learning (DL) can accelerate the prediction of prognostic biomarkers from routine pathology slides in colorectal cancer (CRC). However, current approaches rely on convolutional neural networks (CNNs) and have mostly been validated on small patient cohorts. Here, we develop a new transformer-based pipeline for end-to-end biomarker prediction from pathology slides by combining a pre-trained transformer encoder with a transformer network for patch aggregation. Our transformer-based approach substantially improves the performance, generalizability, data efficiency, and interpretability as compared with current state-of-the-art algorithms. After training and evaluating on a large multicenter cohort of over 13,000 patients from 16 colorectal cancer cohorts, we achieve a sensitivity of 0.99 with a negative predictive value of over 0.99 for prediction of microsatellite instability (MSI) on surgical resection specimens. We demonstrate that resection specimen-only training reaches clinical-grade performance on endoscopic biopsy tissue, solving a long-standing diagnostic problem.

INTRODUCTION

Precision oncology in colorectal cancer (CRC) requires the evaluation of genetic biomarkers, such as microsatellite instability (MSI)[1–8] and mutations in the *BRAF*[4,7] and *NRAS*/*KRAS*[9] genes. These biomarkers are typically assessed by polymerase chain reaction (PCR), sequencing, or immunohistochemical assays. Biomarker identification in patients with CRC is an important

 © 2023 The Author(s). Published by Elsevier Inc.
This is an open access article under the CC BY license (http://creativecommons.org/licenses/by/4.0/).

Matthew Seymour,[20] Laura Magill,[21] Marta Nowak,[22] Jennifer Hay,[23] Viktor H. Koelzer,[22,24,25] David N. Church,[25,26] TransSCOT consortium, Christian Matek,[1,27,28] Carol Geppert,[27,28] Chaolong Peng,[29] Cheng Zhi,[30] Xiaoming Ouyang,[30] Jacqueline A. James,[31,32,33] Maurice B. Loughrey,[33,34,35] Manuel Salto-Tellez,[31,32,36] Hermann Brenner,[8,37,38] Michael Hoffmeister,[8] Daniel Truhn,[39] Julia A. Schnabel,[1,2,40] Melanie Boxberg,[41,42] Tingying Peng,[1,44,*] and Jakob Nikolas Kather[3,5,43,44,45,*]

[19]University Hospital Birmingham, Birmingham, UK
[20]St James's University Hospital, Leeds, UK
[21]University of Birmingham Clinical Trials Unit, Birmingham, UK
[22]Department of Pathology and Molecular Pathology, University Hospital Zurich, University of Zurich, Zurich, Switzerland
[23]Glasgow Tissue Research Facility, University of Glasgow, Queen Elizabeth University Hospital, Glasgow, UK
[24]Department of Oncology, University of Oxford, Oxford, UK
[25]Nuffield Department of Medicine, University of Oxford, Roosevelt Drive, Oxford, UK
[26]Oxford NIHR Comprehensive Biomedical Research Centre, Oxford University Hospitals NHS Foundation Trust, Oxford, UK
[27]Institute of Pathology, University Hospital Erlangen, FAU Erlangen-Nuremberg, Erlangen, Germany
[28]Comprehensive Cancer Center Erlangen-EMN (CCC), University Hospital Erlangen, FAU Erlangen-Nuremberg, Erlangen, Germany
[29]Medical School, Jianggang Shan University, Jiangxi, China
[30]Department of Pathology, the Second Affiliated Hospital of Guangzhou Medical University, Guangzhou, China
[31]Precision Medicine Centre of Excellence, Health Sciences Building, The Patrick G Johnston Centre for Cancer Research, Queen's University Belfast, Belfast, UK
[32]Regional Molecular Diagnostic Service, Belfast Health and Social Care Trust, Belfast, UK
[33]The Patrick G Johnston Centre for Cancer Research, Queen's University Belfast, Belfast, UK
[34]Department of Cellular Pathology, Belfast Health and Social Care Trust, Belfast, UK
[35]Centre for Public Health, Queen's University Belfast, Belfast, UK
[36]Integrated Pathology Unit, Institute for Cancer Research and Royal Marsden Hospital, London, UK
[37]Division of Preventive Oncology, German Cancer Research Center (DKFZ) and National Center for Tumor Diseases (NCT), Heidelberg, Germany
[38]German Cancer Consortium (DKTK), German Cancer Research Center (DKFZ), Heidelberg, Germany
[39]Department of Diagnostic and Interventional Radiology, University Hospital RWTH Aachen, Aachen, Germany
[40]School of Biomedical Engineering and Imaging Sciences, King's College London, London, UK
[41]Institute of Pathology, Technical University Munich, Munich, Germany
[42]Institute of Pathology Munich-North, Munich, Germany
[43]Medical Oncology, National Center for Tumor Diseases (NCT), University Hospital Heidelberg, Heidelberg
[44]These authors contributed equally
[45]Lead contact
*Correspondence: tingying.peng@helmholtz-munich.de (T.P.), jakob_nikolas.kather@tu-dresden.de (J.N.K.)
https://doi.org/10.1016/j.ccell.2023.08.002

step in providing treatment as recommended by various medical guidelines, such as those in the USA (NCCN guideline),[10] UK (NICE guideline),[11] and EU (ESMO guideline).[12] Increasingly, genetic biomarkers such as MSI are also used in earlier tumor stages of CRC.[13] In the future, the importance of biomarker-stratified therapy will likely increase.[14] The presence of MSI should also trigger additional diagnostic processes for a possible diagnosis of Lynch syndrome, one of the most prevalent hereditary cancer syndromes. However, genetic diagnostic assays have several disadvantages. For many patients in low- and middle-income countries, genetic biomarkers are not routinely available due to the prohibitive costs and complex infrastructure required for testing. Even in high-income countries with universal healthcare coverage where genetic biomarkers may be routinely available, their utilization is not without its drawbacks. In such contexts, biomarker assessment can take several days to weeks delaying therapy decisions.[15]

The diagnosis of CRC requires a pathologist's histopathological evaluation of tissue sections. Thus, tissue sections stained with hematoxylin and eosin (H&E) are routinely available for all patients with CRC. Since 2019, dozens of studies have shown that deep learning (DL) can predict genetic biomarkers directly from digitized H&E-stained CRC tissue sections.[1,3,7,8,16,17] Based on these studies, the first commercial DL algorithm for biomarker detection from H&E images has been approved for routine clinical use in Europe in 2022 (MSIntuit, Owkin, Paris/New York).[18] When evaluated in external patient cohorts, the state-of-the-art approaches reach a sensitivity and specificity of 0.95 and 0.46, respectively.[19] Increasing the specificity would be a way to improve these established approaches. Another clinically significant limitation of current approaches is the poor performance on endoscopic biopsy tissue. Recent clinical trials (NICHE[20] and NICHE-2[13]) show high efficacy of neoadjuvant immunotherapy for patients with MSI CRC. These findings imply that in the future every patient with CRC should be tested for MSI on the initial biopsy tissue, although not all current medical guidelines reflect this. Among previous DL-based studies for MSI detection, only Echle et al.[3] determine the performance of DL-based biomarker prediction on CRC biopsy tissue in a multicentric setting. They report a much lower performance on biopsy tissue than on surgical resection tissue sections (biopsy AUROC: 0.78; resection AUROC of 0.96). Current clinically approved commercial products for MSI detection in CRC from histopathology are only applicable to surgical resection tissue. Therefore, DL-based MSI testing of biopsies is a clinical need.

The technology underlying these algorithms in literature is based on weakly supervised learning, consisting of two components: the *feature extractor* and the *aggregation module*.[21] The

feature extractor is mostly based on a convolutional neural network (CNN), which processes multiple small tissue regions called tiles or patches.[22] The CNN-based representations obtained from these tiles are subsequently aggregated to obtain a single prediction for the patient. Between 2019 and 2021, most studies used simple heuristics, such as taking the maximum (max pooling) or averaging (average pooling), as an aggregation module. Since then, variations of multiple instance learning (MIL) have become the new standard for this task, particularly for the prediction of genetic alterations from pathology slides.[6,23,24] The most common approach replaces the pooling aggregation with a small two-layer network to learn the patch-level weighting of the embeddings.[23] However, current MIL approaches univariately consider a single tile during aggregation and do not place it in context with other tiles even though local and global contexts are crucial for medical diagnosis.

In many non-medical and medical image-processing tasks, transformer neural networks have recently been adopted for computer vision tasks,[25–27] replacing CNNs because of their improved performance and robustness.[28] Originally proposed for sequencing tasks such as natural language processing, transformer networks show impressive capabilities of learning long-range dependencies and contextualizing concepts in long sequences. In computational pathology, transformers have therefore been proposed as potentially superior feature extractors[29] or aggregation models,[30–33] though these proposals still lack empirical evidence from large-scale analyses.

In this work, we first aim to enhance the performance of DL-based biomarker detection from pathology slides. Thereafter, in order to provide large-scale evidence of the performance on clinically relevant tasks, we investigate the use of a fully transformer-based workflow in CRC. Here, we present a new method derived from a transformer-based feature extractor and a transformer-based aggregation model (Figure 1A-C), which we evaluate in a large multi-centric study of 15 cohorts with resection specimen slides from over 13,000 patients with CRC worldwide, as well as two cohorts of CRC biopsies from over 1,500 patients in total (Figure 1D-F).

RESULTS

Transformer-based MSI prediction outperforms the state-of-the-art

We tested our pipeline on MSI prediction in surgical resection cohorts of patients with CRC (Figure 1) in two ways: First, we trained the model on a single cohort and tested it on a held-out test set (in-domain) and on all other cohorts (external). We found that large cohorts, e.g., DACHS, QUASAR, or NLCS, achieved in-domain test AUROCs around 0.95 (Figure 2A). The model also achieved high performance close to 0.9 AUROC for early onset CRC, i.e., CRC in patients younger than 50 years (Figure S2B). We compared this performance to the work by Echle et al.[3] which updated the CNN-based feature extractor during training and used mean pooling as their patch aggregation function. Our approach outperformed the CNN-based approach on all four cohorts. Further, we also evaluated AttentionMIL by Ilse et al.[23] with CTransPath as a feature extractor yielding higher performance than the CNN-based approach on the large cohorts but partly lower results on the external validation trained on the smaller cohort TCGA. Overall, we observed the tendency of higher performance and better generalization for models trained on datasets with more than 1,000 patients. However, factors such as differing population genetics (e.g., for MECC) or the type of slide scanners (e.g., for ERLANGEN) influence the generalization capabilities beyond the training dataset size.

Second, we trained our model on all cohorts of CRC resections except YCR-BCIP and evaluated it on the external cohort YCR-BCIP (Figure 2B). In particular, we obtained a sensitivity of 0.99 with a negative predictive value of over 0.99 (Figure S2F, and Table S1). Analyzing the ROCs of patients with different clinicopathological properties showed that the model performs consistently well on all of these subgroups. Only on left-sided tumors the performance slightly dropped to 0.93 AUROC (Figure S2D). Moreover, a high-mean AUPRC score of 0.86 showed that the transformer-based model achieved high sensitivity with high precision despite a strong class imbalance of 12.9% MSI-high samples on average across all cohorts (Figure 2C). In parallel to our findings mentioned previously, we observed a generalization gap when intrinsic biological factors, such as ethnicity, change. However, the performance of our model on a cohort of MSI-high patients from Guangzhou, China, was still high with a sensitivity of 0.9. For a better comparison to state-of-the-art, we also mirrored the experimental setup of Echle et al.[3] We trained AttentionMIL and our fully transformer-based model on the four large cohorts (DACHS, NLCS, QUASAR, and TCGA) using the same feature extractor CTransPath. The CNN-based approach from Echle et al. achieved an AUROC of 0.96, AttentionMIL yielded an AUROC of 0.96, and the fully transformer-based approach performed slightly better with an AUROC of 0.97.

The classical patch-based approach by Echle et al.[3] suffered from severe losses in performance upon external testing. The largest performance drop in the AUROC of 0.21 was observed by a model trained on the DACHS and tested on the QUASAR cohort. Our transformer model, however, reduced the performance loss for external testing to a maximum of 0.09 for training on the NLCS and testing on the TCGA cohort (Figure 2). In addition, AttentionMIL trained with the same transformer-based feature extractor also demonstrated better generalization capabilities compared to the classical patch-based approach with mean pooling. This suggests that self-supervised pre-training on histology data contributes positively toward better generalization.

In summary, these results show that a fully transformer-based approach yields a higher performance for biomarker prediction both on large cohorts (DACHS, QUASAR, and NLCS) as well as on smaller cohorts (TCGA). Perhaps more importantly from a clinical perspective, the transformer-based approach resulted in a better generalization performance and more reliable results, as the deviation between the external cohorts was smaller. We published all trained models for reuse and further fine-tuning if needed.

The transformer-based model predicts multiple biomarkers in CRC

Next, we investigated whether the fully transformer-based model yields a similar high performance in other biomarker-prediction tasks. Following the experimental setup for MSI prediction, we

CellPress
OPEN ACCESS

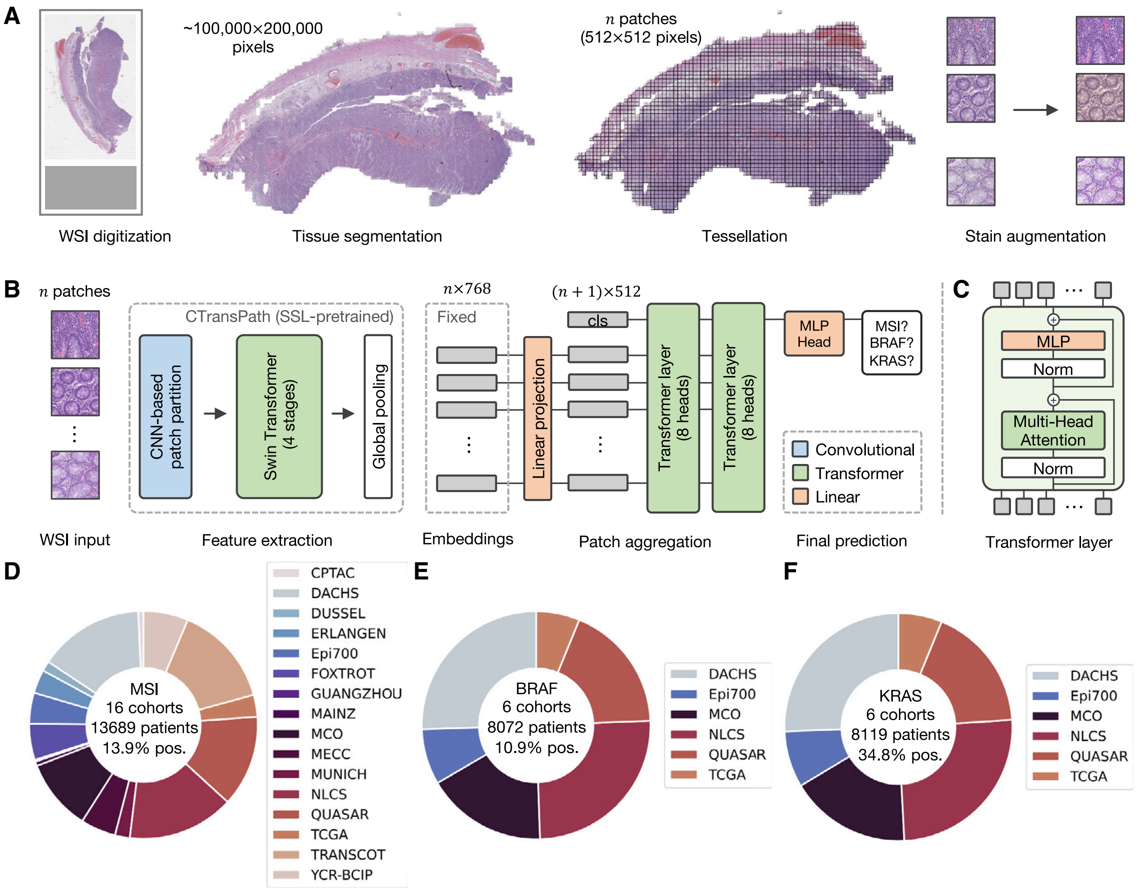

Figure 1. Workflow overview with pre-processing and model architecture and cohort overview

(A) The data pre-processing pipeline with the steps whole slide image (WSI) digitization, tissue segmentation, WSI tessellation into patches, and stain augmentation, (B) the model architecture including the pre-trained feature extractor CTransPath and our transformer-based aggregation module, and (C) details of the transformer layer architecture.

(D) Overview of the 16 cohorts of CRC resections and biopsies with MSI/dMMR status, which were used in this study and the subsets of six cohorts with (E) *BRAF* and (F) *KRAS* ground truth data, respectively.

trained the model first on single cohorts evaluated on all other external cohorts and second on one fully merged multi-center cohort excluding only one cohort from the training set to constitute an external test set. In clinical routine workup for CRC, the biomarkers *BRAF* and *KRAS* are determined in addition to MSI. We tested whether and how well these were predictable on the DACHS, QUASAR, MCO, NLCS, TCGA, and Epi700 cohorts, where the Epi700 cohort served as an external test set in the multi-centric run.

In the case of the largest cohorts, DACHS and NLCS, single cohort training was already capable of achieving good results, with AUROCs of 0.88 and 0.87, respectively (Figure 2D). The smaller cohorts achieved slightly poorer results with 0.83–0.85 AUROC and 0.78 for TCGA. Nonetheless, the AUROC for the in-domain test using TCGA by far outperformed previous approaches with AUROCs of 0.57,[63] 0.66,[64] and 0.73[33] in a more recent transformer-based method. The large multi-centric cohort yielded an AUROC of 0.88, almost reaching clinical-grade performance (Figure 2E). Furthermore, we observed that the generalization gap from the internal test set to external cohorts was consistently small with the largest internal-to-external gap of 0.03 drop in AUROC. This was also the case in multi-centric evaluation, where the performance did not decrease from the internal to the external test cohort.

We observed similar results regarding the generalization when investigating *KRAS* as a target (Figures 2F and 2G), with an AUROC of 0.80 when trained on the multi-centric cohort outperforming state-of-the-art methods. The AUROCs of the single cohort training ranged from 0.53 to 0.77 for single cohorts, in line with or higher than state-of-the-art results.[33,63,64] While DL-based prediction performance for *KRAS* is still relatively low compared to MSI or *BRAF*, the results show that performance profits substantially from multi-cohort training and larger training cohorts.

Overall, these findings show that our model can predict multiple biomarkers that are relevant for routine diagnostics in CRC while highlighting the importance of large training cohorts to reach clinically relevant performance even in biomarkers such as *KRAS* which are notoriously difficult to predict from pathology images alone.

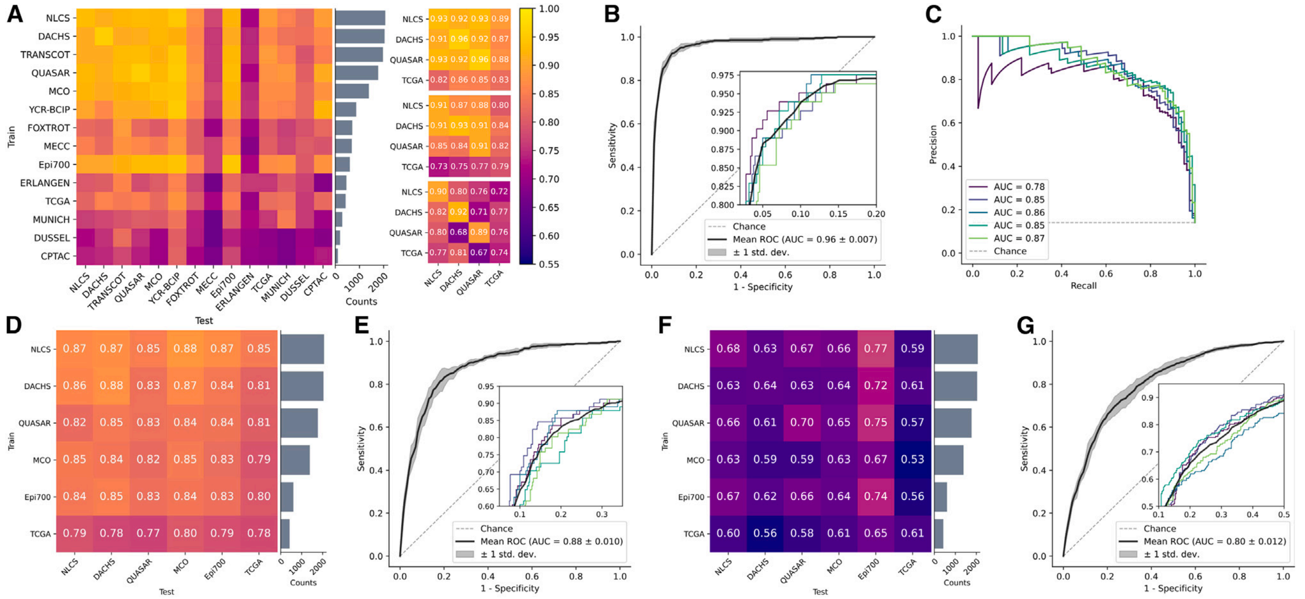

Figure 2. Evaluation of the prediction performance for the biomarkers MSI, *BRAF*, and *KRAS* in single cohort and large-scale multi-centric experiments

Experimental results for MSI-high (A–C), *BRAF* (D,E), and *KRAS* (F,G) prediction. All values represent the mean of 5-fold cross-validation: (A) AUROC scores for single cohort experiments for all CRC cohorts ordered by size of the cohort. Each row shows the test performance of training on one cohort with the in-domain test results in the diagonal. Results for our transformer approach, AttentionMIL, and CNN approach (results taken from Echle et al.) are visualized separately. Note that compared to AttentionMIL and CNN, our transformer not only shows higher overall prediction accuracy but also better model generalizability, demonstrated by a smaller gap between internal and external testing cohorts. Raw data for the heatmap in Table S5.

(B) Receiver operator curve (ROC) for the model trained on all resection cohorts except YCR-BCIP, tested on YCR-BCIP.

(C) Precision recall curve (PRC) for the model trained on all resection cohorts except YCR-BCIP, tested on YCR-BCIP.

(D) AUROC scores for single cohort experiments.

(E) ROC for the model trained on all *BRAF* cohorts except Epi700, tested on Epi700.

(F) AUROC scores for single cohort experiments.

(G) ROC for the model trained on all *KRAS* cohorts except Epi700, tested on Epi700.

Transformer-based workflows are explainable

Ideally, DL-based biomarker predictions should be explainable to domain experts. To this end, we visualized how much each patch contributed to the final classification via attention rollout as well as whether it contributes toward a positive or negative classification (Figures 3A–3C, and S3).

For better comparability, we used the same WSIs from the external cohort YCR-BCIP as had been used in a previous study[3] for these visualizations (Figure 3A). For all three cases, the majority of highly contributing patches originate from tumor regions. In the MSI-high case, the mucinous region that is morphologically linked to MSI is correctly identified as important by high scores in the attention rollout as well as the patch-level classification (see the boxes in the first row of Figure 3). The MS-stable case in the second row of Figures 3A–3C attributes high contributions to the model's prediction to carcinoma regions. At the same time, these patches all receive low-classification scores yielding the correct classification result. Similarly, the second MS-stable case in the third row had highly contributing scores in the tumor region while having only low-classification scores for all patches. Further tissue details that are morphologically related to MSI, such as solid growth pattern, poor differentiation, or tumor-infiltrating lymphocytes (Figure S3A) are highlighted in the attention heads of the last layer together with healthy tissue structures, such as the colon wall including muscle tissue or vessels (Figure 3D). The two cases with MSI-high ground truth predicted as MSS also show that relevant regions are identified and contribute to the prediction but the combination of potentially false attentions and associated classification scores of these attentive patches infer a wrong prediction (Figure S3B).

We quantified the morphological patterns occurring in high-attention regions in a small user study, where two pathologists annotated the patterns in 160 patches of 40 patients that the model attributes high attention to. We chose the 10 patients with the lowest and highest classification score for each MSS and MSI-high ground truth. For every patient, we chose the two patches associated with the highest and lowest class scores of the top 100 attention tiles. Our study showed that the majority of tiles belong to the tumor region (0.99% for high and 0.81% for low-classification scores) and cell types that are important for the prediction of MSI-high, such as lymphocytes occur in both tiles with low- and high-classification score in a similar ratio (0.28% vs. 0.2%, Figure 4A). Furthermore, morphological patterns related only to MSI-high, such as mucinous regions, occur more often in tiles with high-classification scores (0.4% vs. 0.1%). A chi-square test for independence shows that the underlying distributions of tiles with high- and low-classification scores are likely to be independent (p value = $5.6 \cdot 10^{-6} < 5 \cdot 10^{-5}$).

CellPress
OPEN ACCESS

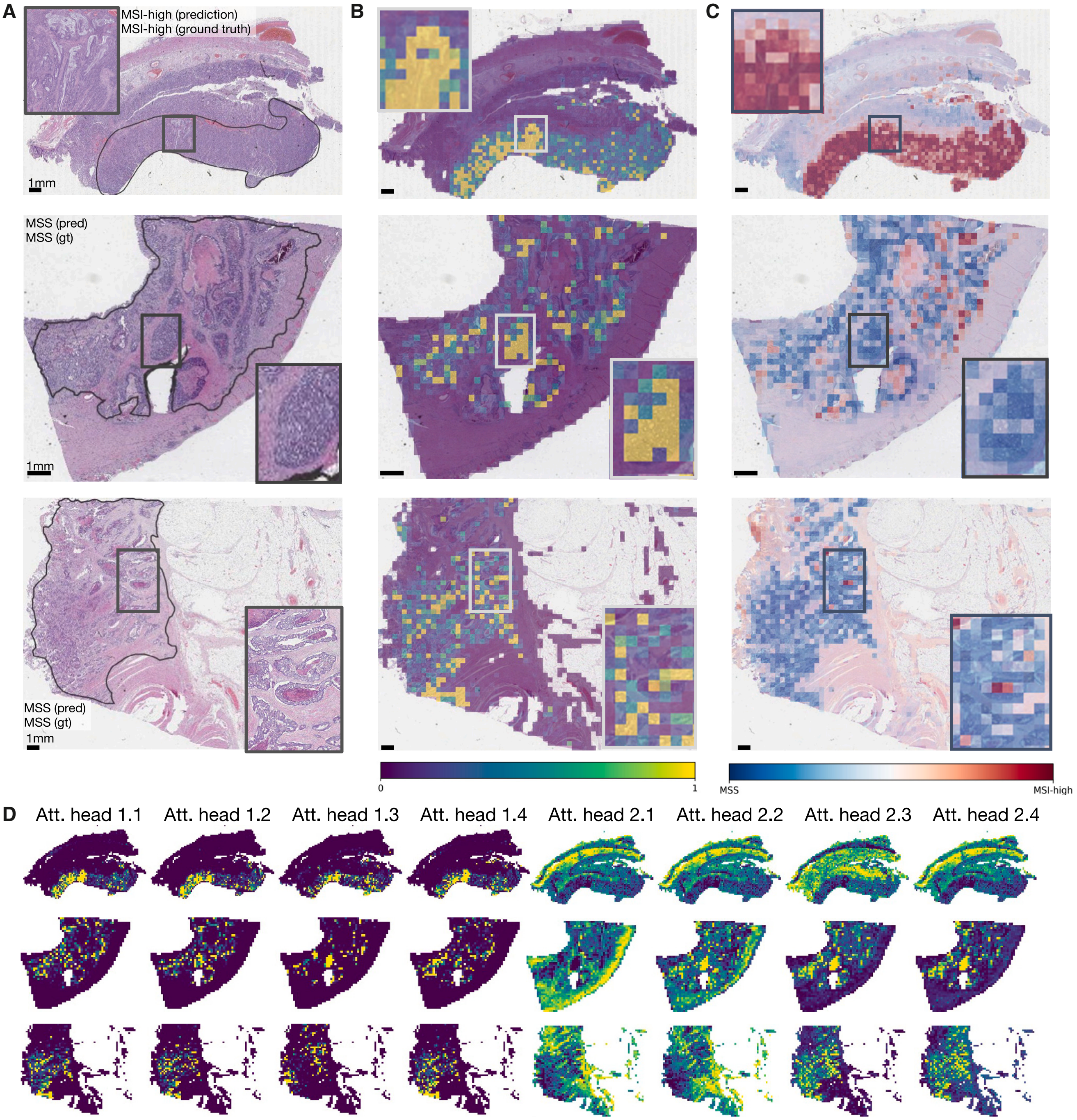

Figure 3. Attention and class score visualization for better model interpretability

(A) Resection specimen from the external cohort YCR-BCIP. The three depicted slides are the same as in Echle et al.[3] Tumor regions are outlined in black.

(B) Attention rollout per patch for our trained transformer-based feature aggregation model. Large values (yellow) signify a high contribution to the model's prediction, small values (purple) a low contribution.

(C) MSI classification scores per patch, where MSI-high is the positive class and MS-stable is the negative class.

(D) The attention heatmaps from eight heads, four of the first and four of the second layer. The model weights are taken from the best-performing fold of the multicentric training on all cohorts except YCR-BCIP.

These examples showed that the model learns concepts relevant to MSI-high prediction and thus possesses a high degree of explainability. Visualizing the attention rollout together with the classification scores demonstrates that relevant regions can receive high-attention scores while the model can learn to ignore non-relevant regions or give them low-classification scores.

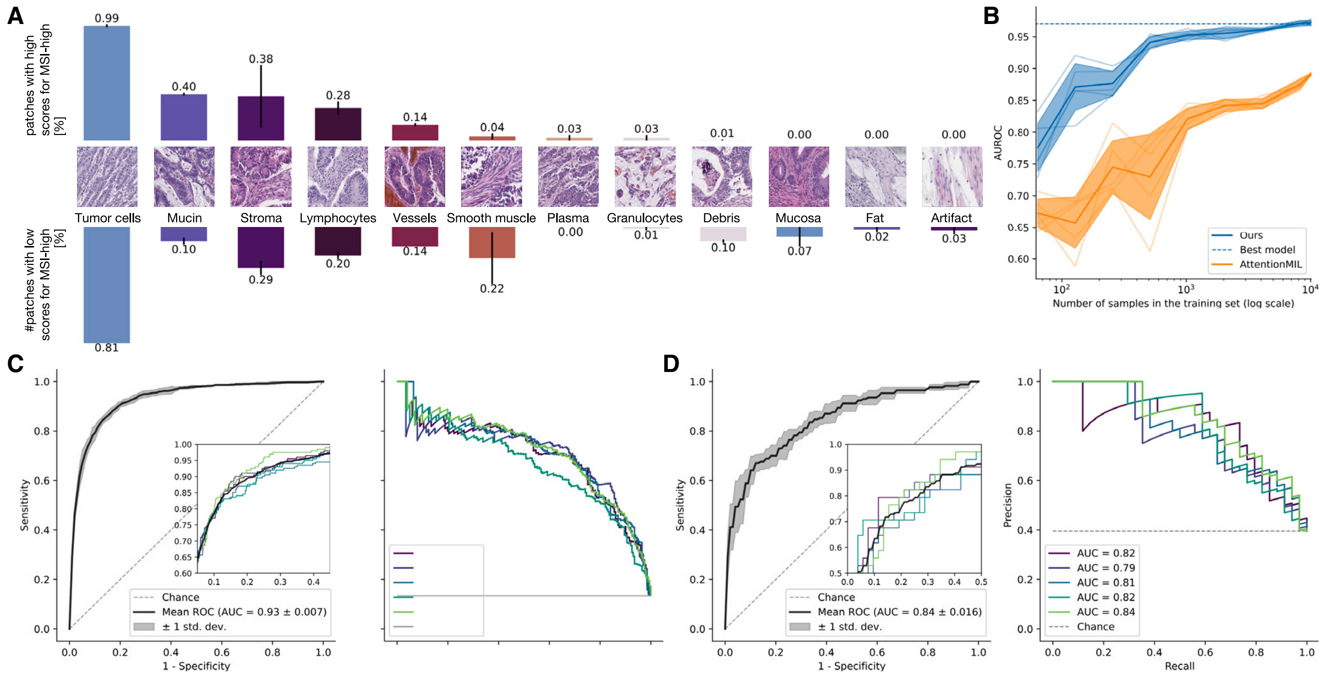

Figure 4. Analysis of the quantitative user study on high-attention tiles, data efficiency, and model generalization to biopsies

(A) Prevalence of 12 histological patterns in 160 patches of high attention regions from 40 patients split by low and high patch-wise classification scores.

(B) AUROC scores on YCR-BCIP depending on the number of patients available for training. The samples were randomly drawn from all resection cohorts except YCR-BCIP.

(C) ROC and PRC for testing our model on YCR-BCIP-biopsies, trained on resections from all cohorts except YCR-BCIP.

(D) ROC and PRC for testing our model on biopsies of the cohort MAINZ, trained on resections from all cohorts except YCR-BCIP.

Transformer-based workflows are more data efficient

A long-standing problem in computational pathology is to determine the number of samples required for a given prediction task. This is primarily due to two reasons. First, it is unclear what the minimum required sample size is, and second, it is unclear if adding more samples improves performance, and if so, up to what point. To investigate this, we varied the number of patients in the training set and analyzed its impact on the test performance. Specifically, we merged all cohorts except for an external validation cohort, YCR-BCIP, resulting in a training set with 8,181 patients from nine cohorts. We trained models using a fixed number of epochs and randomly sampled patients from the training set. All experiments were repeated five times, and we reported the means and standard deviations of the results.

Our fully transformer-based model architecture achieved a mean testing AUROC value above 0.9 with 250 patients (in particular, an AUROC value of 0.92), while the AttentionMIL model exceeded an AUROC of 0.9 only with 4,000 patients (Figure 4A). In a similar vein, our model surpassed the 0.95 mean testing AUROC with already 1,500 patients, while AttentionMIL did not reach this performance. Hence, the transformer-based aggregation module helped the model to learn from data in a more efficient way than the attention mechanism. This may be due to the attention mechanism not contextualizing information from all input patches. Of note, above 1,000 patients, the performance of the transformer-based model seems to slowly saturate, while the attention mechanism continues to increase in performance with more patients but on a lower level.

Our fully transformer-based approach yielded high performance with a small sample size. Compared to an AttentionMIL-based approach, our approach is more data efficient in the regime of small numbers of patients. Looking at larger training numbers, we observed that performance increase is directly proportional to the number of patients for both approaches, but the fully transformer-based approach reaches equivalent performance already with much smaller datasets.

Transformer-based workflows result in clinical-grade performance on biopsies

Virtually all previous studies on biomarker prediction in CRC were performed using surgical resection slides. For this reason, commercially available MSI detection algorithms are intended to be used only with resection slides. However, recent clinical evidence shows that MSI-positive patients with CRC require immunotherapy prior to surgery[13,65] and hence need to be tested for MSI on biopsy material. We addressed this problem by training our model on resections from all cohorts except YCR-BCIP and evaluating it on biopsies from 1,592 patients with CRC of the YCIP-BCIP.

Our model yielded a mean AUROC score of 0.92 and 0.86 when validated on biopsies from two external cohorts, YCR-BCIP and MAINZ, respectively (Figure 4B). It is worth mentioning that the MSI-high ratio in the MAINZ biopsy cohort was higher than in the training cohorts. We outperformed existing approaches (0.78 by Echle et al.[3]) by far and achieved clinical-grade performance on biopsies after model training on resection

CellPress
OPEN ACCESS

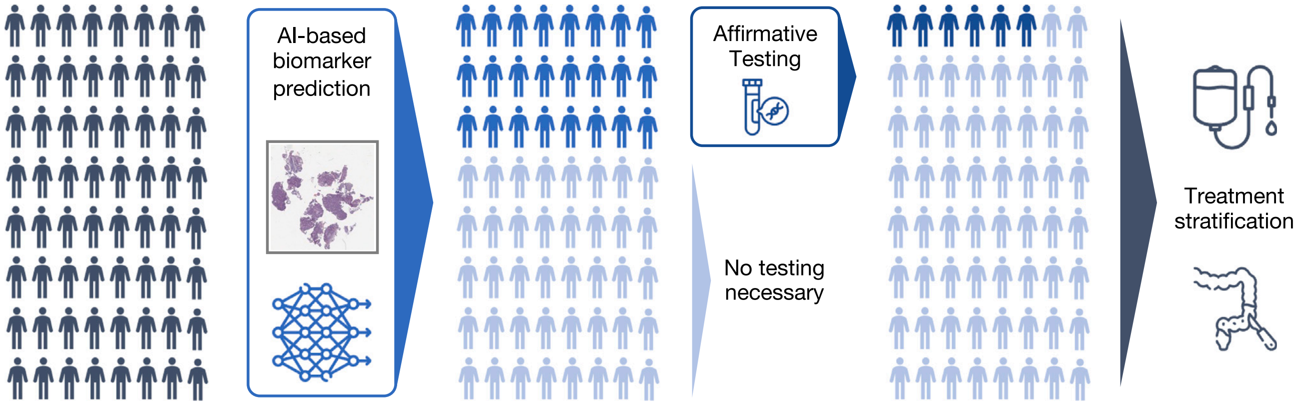

Figure 5. Envisioned clinical workflow for the proposed MSI-high classifier on biopsies

This assumes the system reaches a sufficient performance in additional external validation and is approved as a medical device. This workflow would only apply to non-metastatic disease. Neoadjuvant immunotherapy is not yet recommended by medical guidelines but is backed up by Phase-II clinical trials. Not shown: tissue preprocessing and scanning pipelines and confirmatory tests of MSI-high after a positive deep learning-based pre-screening.

specimens (Figure S4). The mean AUPRC score of 0.69 and 0.82, respectively, however, was lower than that of the external cohort YCR-BCIP for resections (0.86) (Figure 4C). Hence, choosing a classification threshold with high sensitivity, the ratio of correctly MSI positive predicted cases from all positive predicted cases was lower on biopsies compared to resections. Still, with a classification threshold fixed on the in-domain test set of resections, our model obtains sensitivity scores of 0.98 and 0.91, respectively, with negative predictive values of 0.99 and 0.9. Of note, these values are higher than (for the cohort YCR-BCIP) and close to (for the MAINZ cohort) the clinically approved DL algorithm for resections,[19] suggesting that our algorithm has potential for clinical usage for biopsies.

Our intended clinical use of this workflow is as follows (Figure 5): First, a patient attends a clinic either with suspected CRC or for routine CRC screening. A colonoscopy shows a suspicious tumor, which is evaluated histologically and found to be an adenocarcinoma. In many countries, this biopsy will then be tested for MSI/MMR status and *BRAF* and/or *RAS* mutation status. In practice, these procedures may take several days to even weeks. However, in low- or middle-income countries, this might not happen at all. Based on the MSI, *BRAF*, and *RAS* status, the most suitable treatment approach will be chosen for the patient. For example, in patients with early (non-metastatic) CRC, the presence of MSI could qualify a patient for neoadjuvant immunotherapy followed by surgery with curative intent. Similarly, in the metastatic disease setting, the presence of MSI in the biopsy tissue would qualify a patient for palliative immunotherapy. Because of its high sensitivity, our algorithm could serve as a filtering step followed by affirmative testing for MSI-high predicted cases. Applying AI-based biomarker prediction would reduce the additional testing burden and therefore speed up the step between taking the biopsy and the molecular determination of MSI-high status, thus enabling an earlier treatment with immunotherapy if indicated.

In summary, to the best of our knowledge, we developed a DL-based MSI-high predictor for biopsies that achieves clinical-grade performance. In particular, this high performance was also observed for external tests and could therefore improve clinical routine and speed up treatment decisions.

DISCUSSION

The rollout of precision oncology to patients with CRC promises gains in life expectancy.[66] Unfortunately, however, its implementation still remains slow and patchy. One reason for this is that precision oncology biomarkers are complex, costly, and require intricate instrumentation and expertise. DL is emerging as a possible solution for this problem.[22,67] DL can extract biomarker information directly from routinely available material, thereby potentially providing cost savings.[1] Using DL-based analysis of histopathology slides to extract biomarkers for oncology has become a common approach in the research setting in 2018.[68] In turn, this has recently led to regulatory approval of multiple algorithms for clinical use. Some of these examples include a breast cancer survival prediction algorithm by Paige (New York, NY, USA), a method to predict survival in CRC by DoMore Diagnostics (Oslo, Norway), a method to predict MSI status in CRC by Owkin (Paris, France, and New York, NY, USA), among others.[18,69] However, existing DL biomarkers have some key limitations: it is debated whether or not their performance is sufficient for large-scale use, they do not necessarily generalize to any patient population, and finally, they are not approved for use on biopsy material, as the application of DL algorithms to biopsies typically results in much lower performance compared to application to surgical specimens.[3]

A key reason for the limited performance of existing DL systems could be the fundamental limitations of the technology employed. Most studies between 2018 and 2020 used convolutional neural networks (CNNs) as their DL backbone,[31] using publicly available information. Commercial products in the DL biomarker space are based on the same technology.[19,69,70] However, a new class of neural networks has recently started to replace CNNs: transformers. Originating from the field of natural language processing, transformers are a powerful tool to process sequences and leverage the potential of large amounts of data. Also in computer vision, transformers yield a higher accuracy for image classification in non-medical tasks,[25,26] are more robust to distortions in the input data[28] and provide more detailed explainability.[30] These advantages of transformers

compared to CNNs have the potential to translate into more accurate and more generalizable clinical biomarkers, but there is currently no evidence to support this.

In the present study, we developed a transformer-based approach for biomarker prediction on whole-slide images of H&E-stained CRC tissue sections. Our model consists of a transformer-based feature extractor that was pretrained on histopathology images and a transformer-based aggregation module. In contrast to the state-of-the-art attention-based MIL approaches, the contribution of each patch was not only determined according to its feature embeddings but also contextualized with the feature embeddings of all other patches in the WSI via self-attention layers. Further, we presented a large-scale evaluation of transformers in biomarker prediction on WSIs. We demonstrated that transformer-based approaches learned better from small amounts of data and were therefore more data efficient than attention-based MIL approaches. At the same time, the performance increased proportionally with the number of training samples. Even though the performance seemed to plateau for MSI prediction, this suggests that larger training cohorts could lead to higher performance-approaching clinical application, also for more challenging tasks such as the prediction of the *BRAF* and *KRAS* mutational status. Our large-scale evaluation also showed that MIL and in particular transformer-based approaches generalize much better than the existing CNN approaches. We proved this by training the model on single cohorts and testing the generalization on all other cohorts. These experiments showed that the transformer-based approach reduced the drop in AUROC to under 0.09, while the CNN-based approach dropped by more than 0.21 in some cohorts. Most importantly, our approach trained on resections did not only generalize well to external cohorts of resections from geographically distinct regions but also to biopsies with a clinical-grade performance of 0.98 sensitivity on the YCR-BCIP biopsy cohort and 0.91 on the MAINZ cohort.

A caveat of our observations is that the ground truth might not be perfect. Potentially, the DL model is performing better than stated in the paper because dMMR and MSI only agree around 92% of the time and neither are 100% sensitive.[57,71] Also, a small subset of CRCs have *POLD1* and *POLE* mutations with a high-mutation burden that behaves clinically similar to MSI and might have a similar phenotype but are not detected by established MSI detection assays.[72] Similarly, gene sequencing does not detect all mutations in *KRAS*/*NRAS*/*BRAF* depending on the sensitivity of the technology used and the presence of smaller clonal mutations. Current DL tests are at such high levels of performance that these nuanced subpopulations may be important. Our study has additional limitations: The focus of this study was to investigate the effect of handling the data with fully transformer-based approaches, especially in the context of large-scale multi-institutional data. Therefore, we did not exhaustively optimize every single hyperparameter. Points for optimization in this direction would be finding a fitting positional encoding and tuning the architecture of the transformer network and attention mechanisms. Additionally, collecting biopsy samples from different hospitals, for multi-cohort training directly on biopsy data could potentially improve the performance of our model on biopsy material. This would also hold for the prediction of *BRAF* mutation status and, in particular, of *RAS* mutation status, where we observed the largest potential for improvement. In both targets, the performance was higher on the larger cohorts with around 2,000 patients and increased dramatically by training on multiple cohorts. Further, we acknowledge that achieving an even higher specificity would be desirable. Choosing the final classification threshold is always a trade-off between sensitivity and specificity, where clinical application prefers a higher sensitivity, especially for pre-screening test as proposed in this study. Our method's performance on biopsies is in the same range as current clinically approved assays on resections, but unlike these assays, our method also works on biopsies.

In summary, to the best of our knowledge, we presented a fully transformer-based model to predict MSI on WSI from CRC with an AUROC of 0.97 on resections and 0.92 and 0.86 on biopsies on external validation cohorts. Our model generalized better to unseen cohorts and was more data efficient compared to existing state-of-the-art MIL or CNN approaches. By publishing all trained models, we enable researchers and clinicians to apply the automated MSI prediction tool for research purposes, which we expect to bring the field of DL-based biomarkers a step closer to large-scale integration in the clinical workflow.

STAR★METHODS

Detailed methods are provided in the online version of this paper and include the following:

- KEY RESOURCES TABLE
- RESOURCE AVAILABILITY
  - Lead contact
  - Materials availability
  - Data and code availability
- EXPERIMENTAL MODEL AND STUDY PARTICIPANT DETAILS
  - Ethics statement
  - Cohort description
- METHOD DETAILS
  - Model description
  - Experimental setup and implementation details
  - Visualization and explainability
- QUANTIFICATION AND STATISTICAL ANALYSIS

SUPPLEMENTAL INFORMATION

Supplemental information can be found online at https://doi.org/10.1016/j.ccell.2023.08.002.

CONSORTIA

We acknowledge the support of the Rainbow-TMA Consortium, especially the project group: PA van den Brandt, A zur Hausen, HI Grabsch, M van Engeland, LJ Schouten, J Beckervordersandforth; PHM Peeters, PJ van Diest, HB Bueno de Mesquita; J van Krieken, I Nagtegaal, B Siebers, B Kiemeney; FJ van Kemenade, C Steegers, D Boomsma, GA Meijer; FJ van Kemenade, B Stricker; L Overbeek, A Gijsbers; and Rainbow-TMA collaborating pathologists, among others: A de Bruïne; JC Beckervordersandforth; J van Krieken, I Nagtegaal; W Timens; FJ van Kemenade; MCH Hogenes; PJ van Diest; RE Kibbelaar; AF Hamel; ATMG Tiebosch; C Meijers; R Natté; GA Meijer; JJTH Roelofs; RF Hoedemaeker; S Sastrowijoto; M Nap; HT Shirango; H Doornewaard; JE

CellPress
OPEN ACCESS

Boers; JC van der Linden; G Burger; RW Rouse; PC de Bruin; P Drillenburg; C van Krimpen; JF Graadt van Roggen; SAJ Loyson; JD Rupa; H Kliffen; HM Hazelbag; K Schelfout; J Stavast; I van Lijnschoten; and K Duthoi.

ACKNOWLEDGMENTS

SJW and DR are supported by the Helmholtz Association under the joint research school "Munich School for Data Science - MUDS" and SJW is supported by the Add-on Fellowship of the Joachim Herz Foundation. JNK is supported by the German Federal Ministry of Health (DEEP LIVER, ZMVI1-2520DAT111), the Max-Eder-Programme of the German Cancer Aid (grant #70113864), the German Federal Ministry of Education and Research (CAMINO, 01EO2101; SWAG, 01KD2215A; TRANSFORM LIVER, 031L0312A; TANGERINE, 01KT2302 through ERA-NET Transcan), the German Academic Exchange Service (SECAI, 57616814), the German Federal Joint Committee (Transplant.KI, 01VSF21048) and the European Union's Horizon Europe and innovation program (ODELIA, 101057091; GENIAL, 101096312). JNK and MH are funded by the German Federal Ministry of Education and Research (PEARL, 01KD2104C). PQ, NW, SD, and GH are supported by Yorkshire Cancer Research grants L386 and L394. PQ, HG, NW, JNK, and SD are supported in part by the National Institute for Health and Care Research (NIHR) Leeds Biomedical Research Center. The views expressed are those of the author(s) and not necessarily those of the NHS, the NIHR, or the Department of Health and Social Care. PQ is also supported by an NIHR Senior Investigator award. FOxTROT was funded by Cancer Research UK (grant reference: C551/A8283; recipient: D.M.). Additional support was provided by the Birmingham and Leeds ECMC network, the RCS Eng and Rosetrees Trust, and the Swedish Cancer Society. Panitumumab was provided free of charge by Amgen, who also supported *RAS* testing and additional CT scans (recipient: D.M.). P.Q., N.W., and M.S. are supported by Yorkshire Cancer Research, R.G. by the Medical Research Council. Tumor tissue collection in the NLCS was done in the Rainbow-TMA study, which was financially supported by BBMRI-NL, a Research Infrastructure financed by the Dutch government (NWO 184.021.007 to PvdB). The analyses of MSI, *BRAF,* and *KRAS* in the NLCS were funded by The Dutch Cancer Society (KWF 11044 to PvdB). The DACHS study (HB, JCC, and MH) was supported by the German Research Council (BR 1704/6-1, BR 1704/6-3, BR 1704/6-4, CH 117/1-1, HO 5117/2-1, HO 5117/2-2, HE 5998/2-1, HE 5998/2-2, KL 2354/3-1, KL 2354/3-2, RO 2270/8-1, RO 2270/8-2, BR 1704/17-1 and BR 1704/17-2), the Interdisciplinary Research Program of the National Center for Tumor Diseases (NCT; Germany) and the German Federal Ministry of Education and Research (01KH0404, 01ER0814, 01ER0815, 01ER1505A and 01ER1505B). The study was further supported by project funding for the Pearl consortium from the German Federal Ministry of Education and Research (01KD2104A). SF is supported by the Deutsche Forschungsgemeinschaft (DFG) (FO 942/2-1), the German Federal Ministry of Education and Research (SWAG, 01KD2215A), the Mainz Research School of Translational Biomedicine (TransMed) and the Manfred-Stolte-Foundation. CM is supported by the Interdisciplinary Center for Clinical Research (IZKF) at the University Hospital of the University of Erlangen-Nuremberg (Junior Project J101). This work was supported in part by NIH R01 CA263318 (SG).

DACHS study: The authors thank the hospitals recruiting patients for the DACHS study and the cooperating pathology institutes. We thank the National Center for Tumor Diseases (NCT) Tissue Bank, Heidelberg, Germany, for managing, archiving, and processing tissue samples in the DACHS study.

The SCOT trial was funded by the Medical Research Council (transferred to NETSCC - Efficacy and Mechanism Evaluation) (Grant Ref: G0601705), NIHR Health Technology Assessment (Grant ref. 14/140/84), Cancer Research UK Core CTU Glasgow Funding (Funding Ref: C6716/A9894), and the Swedish Cancer Society. The TransSCOT sample collection was funded by a Cancer Research UK Clinical Trials Awards and Advisory Committee – Sample Collection (Grant Ref: C6716/A13941).

Molecular analysis of the SCOT samples were funded by the Oxford NIHR Comprehensive Biomedical Research Centre (BRC), a Cancer Research UK (CRUK) Advanced Clinician Scientist Fellowship (C26642/A27963) to DNC, CRUK award A25142 to the CRUK Glasgow Center. V.H.K. acknowledges funding by the Preeclampsia Foundation (F-87701-41-01). The views expressed are those of the authors and not necessarily those of the NHS, the NIHR, the Department of Health.

We furthermore acknowledge all collaborators in the YCR-BCIP and FOxTROT studies, as well as all other collaborators for all other cohorts included in this study.

AUTHOR CONTRIBUTIONS

S.J.W., M.B., T.P., and J.N.K. designed the concept of the study, S.J.W., J.M.N., and J.Z. prepared the data for this study, S.J.W. implemented the methods and ran all experiments and evaluations; S.F., C.M., C.P., M.B., and G.P.V. supported with domain-related advice; D.R., S.J.W., M.B., and T.P. developed and evaluated a preliminary study, where D.R. implemented the methods; all authors provided clinical and histopathological data and expertise; all authors provided clinical expertise and contributed to the interpretation of the results. S.J.W. wrote the manuscript with J.N.K. and input from all other authors.

DECLARATION OF INTERESTS

J.N.K. reports consulting services for Owkin, France, Panakeia, UK, and DoMore Diagnostics, Norway and has received honoraria for lectures by M.S.D., Eisai, and Fresenius. N.W. has received fees for advisory board activities with BMS, Astellas, GSK, and Amgen, not related to this study. N.W. has received fees for advisory board activities with BMS, Astellas, and Amgen, not related to this study. P.Q. has received fees for advisory board activities with Roche and AMGEN and research funding from Roche through an Innovate UK National Pathology Imaging Consortium grant. H.I.G. has received fees for advisory board activities by AstraZeneca and BMS, not related to this study. M.S.T. is a scientific advisor to Mindpeak and Sonrai Analytics, and has received honoraria recently from BMS, MSD, Roche, Sanofi, and Incyte. He has received grant support from Phillips, Roche, MSD, and Akoya. None of these disclosures are related to this work. D.N.C. has participated in advisory boards for MSD and has received research funding on behalf of the TransSCOT consortium from HalioDx for analyses independent of this study. V.H.K. has served as an invited speaker on behalf of Indica Labs and has received project-based research funding from The Image Analysis Group and Roche outside of the submitted work. No other potential disclosures are reported by any of the authors.

Received: January 4, 2023
Revised: June 18, 2023
Accepted: August 7, 2023
Published: August 30, 2023

REFERENCES

1. Kather, J.N., Pearson, A.T., Halama, N., Jäger, D., Krause, J., Loosen, S.H., Marx, A., Boor, P., Tacke, F., Neumann, U.P., et al. (2019). Deep learning can predict microsatellite instability directly from histology in gastrointestinal cancer. Nat. Med. *25*, 1054–1056.
2. Cao, R., Yang, F., Ma, S.-C., Liu, L., Zhao, Y., Li, Y., Wu, D.-H., Wang, T., Lu, W.-J., Cai, W.-J., et al. (2020). Development and interpretation of a pathomics-based model for the prediction of microsatellite instability in Colorectal Cancer. Theranostics *10*, 11080–11091.
3. Echle, A., Grabsch, H.I., Quirke, P., van den Brandt, P.A., West, N.P., Hutchins, G.G.A., Heij, L.R., Tan, X., Richman, S.D., Krause, J., et al. (2020). Clinical-Grade Detection of Microsatellite Instability in Colorectal Tumors by Deep Learning. Gastroenterology *159*, 1406–1416.e11.
4. Bilal, M., Ahmed Raza, S.E., Azam, A., Graham, S., Ilyas, M., Cree, I.A., Snead, D., Minhas, F., and Rajpoot, N.M. Novel Deep Learning Algorithm Predicts the Status of Molecular Pathways and Key Mutations in Colorectal Cancer from Routine Histology Images. 10.1101/2021.01.19.21250122
5. Lee, S.H., Song, I.H., and Jang, H.-J. (2021). Feasibility of deep learning-based fully automated classification of microsatellite instability in tissue slides of colorectal cancer. Int. J. Cancer *149*, 728–740.
6. Schirris, Y., Gavves, E., Nederlof, I., Horlings, H.M., and Teuwen, J. (2022). DeepSMILE: Contrastive self-supervised pre-training benefits MSI and

HRD classification directly from H&E whole-slide images in colorectal and breast cancer. Med. Image Anal. *79*, 102464.

7. Schrammen, P.L., Ghaffari Laleh, N., Echle, A., Truhn, D., Schulz, V., Brinker, T.J., Brenner, H., Chang-Claude, J., Alwers, E., Brobeil, A., et al. (2022). Weakly supervised annotation-free cancer detection and prediction of genotype in routine histopathology. J. Pathol. *256*, 50–60.
8. Yamashita, R., Long, J., Longacre, T., Peng, L., Berry, G., Martin, B., Higgins, J., Rubin, D.L., and Shen, J. (2021). Deep learning model for the prediction of microsatellite instability in colorectal cancer: a diagnostic study. Lancet Oncol. *22*, 132–141.
9. Jang, H.-J., Lee, A., Kang, J., Song, I.H., and Lee, S.H. (2020). Prediction of clinically actionable genetic alterations from colorectal cancer histopathology images using deep learning. World J. Gastroenterol. *26*, 6207–6223.
10. Benson, A.B., Venook, A.P., Al-Hawary, M.M., Cederquist, L., Chen, Y.-J., Ciombor, K.K., Cohen, S., Cooper, H.S., Deming, D., Engstrom, P.F., et al. (2018). NCCN Guidelines Insights: Colon Cancer, Version 2.2018. J. Natl. Compr. Cancer Netw. *16*, 359–369.
11. National Institute for Health and Care Excellence (2020). Colorectal cancer [NICE Guideline No. 151]. https://www.nice.org.uk/guidance/ng151.
12. Schmoll, H.J., Van Cutsem, E., Stein, A., Valentini, V., Glimelius, B., Haustermans, K., Nordlinger, B., van de Velde, C.J., Balmana, J., Regula, J., et al. (2012). ESMO Consensus Guidelines for management of patients with colon and rectal cancer. a personalized approach to clinical decision making. Ann. Oncol. *23*, 2479–2516.
13. Chalabi, M., Verschoor, Y.L., van den Berg, J., Sikorska, K., Beets, G., Lent, A.V., Grootscholten, M.C., Aalbers, A., Buller, N., Marsman, H., et al. (2022). LBA7 Neoadjuvant immune checkpoint inhibition in locally advanced MMR-deficient colon cancer: The NICHE-2 study. Ann. Oncol. *33*, S1389.
14. Vacante, M., Borzì, A.M., Basile, F., and Biondi, A. (2018). Biomarkers in colorectal cancer: Current clinical utility and future perspectives. World J. Clin. Cases *6*, 869–881.
15. Lim, C., Tsao, M.S., Le, L.W., Shepherd, F.A., Feld, R., Burkes, R.L., Liu, G., Kamel-Reid, S., Hwang, D., Tanguay, J., et al. (2015). Biomarker testing and time to treatment decision in patients with advanced non-small-cell lung cancer. Ann. Oncol. *26*, 1415–1421.
16. Niehues, J.M., Quirke, P., West, N.P., Grabsch, H.I., van Treeck, M., Schirris, Y., Veldhuizen, G.P., Hutchins, G.G.A., Richman, S.D., Foersch, S., et al. (2023). Generalizable biomarker prediction from cancer pathology slides with self-supervised deep learning: A retrospective multi-centric study. Cell Rep. Med. *4*, 100980.
17. Bilal, M., Raza, S.E.A., Azam, A., Graham, S., and Ilyas, M. (2021). Development and validation of a weakly supervised deep learning framework to predict the status of molecular pathways and key mutations in colorectal …. The Lancet Digital.
18. Saillard, C., Dubois, R., Tchita, O., Loiseau, N., Garcia, T., Adriansen, A., Carpentier, S., Reyre, J., Enea, D., Kamoun, A., et al. (2022). Blind validation of MSIntuit, an AI-based pre-screening tool for MSI detection from histology slides of colorectal cancer. Preprint at medRxiv.
19. Svrcek, M., Saillard, C., Dubois, R., Loiseau, N., Mespoulhe, P., Brulport, F., Guillon, J., Auffret, M., Sefta, M., Kamoun, A., et al. (2022). 920P Blind validation of MSIntuit, an AI-based pre-screening tool for MSI detection from colorectal cancer H&E slides. Ann. Oncol. *33*, S967.
20. Chalabi, M., Fanchi, L.F., Dijkstra, K.K., Van den Berg, J.G., Aalbers, A.G., Sikorska, K., Lopez-Yurda, M., Grootscholten, C., Beets, G.L., Snaebjornsson, P., et al. (2020). Neoadjuvant immunotherapy leads to pathological responses in MMR-proficient and MMR-deficient early-stage colon cancers. Nat. Med. *26*, 566–576.
21. Bilal, M., Jewsbury, R., Wang, R., AlGhamdi, H.M., Asif, A., Eastwood, M., and Rajpoot, N. (2022). An Aggregation of Aggregation Methods in Computational Pathology. Preprint at arXiv. [cs.CV].
22. Shmatko, A., Ghaffari Laleh, N., Gerstung, M., and Kather, J.N. (2022). Artificial intelligence in histopathology: enhancing cancer research and clinical oncology. Nat. Can. (Ott.) *3*, 1026–1038.
23. Ilse, M., Tomczak, J., and Welling, M. (10–15 Jul 2018) Attention-based Deep Multiple Instance Learning. In Proceedings of the 35th International Conference on Machine Learning Proceedings of Machine Learning Research., J. Dy and A. Krause, eds. (PMLR), pp. 2127–2136
24. Saldanha, O.L., Loeffler, C.M.L., Niehues, J.M., van Treeck, M., Seraphin, T.P., Hewitt, K.J., Cifci, D., Veldhuizen, G.P., Ramesh, S., Pearson, A.T., and Kather, J.N. (2023). Self-supervised attention-based deep learning for pan-cancer mutation prediction from histopathology. npj Precis. Oncol. *7*, 35.
25. Dosovitskiy, A., Beyer, L., Kolesnikov, A., Weissenborn, D., Zhai, X., Unterthiner, T., Dehghani, M., Minderer, M., Heigold, G., Gelly, S., et al. (2020). An Image is Worth 16x16 Words: Transformers for Image Recognition at Scale. Preprint at arXiv. [cs.CV].
26. Liu, Z., Lin, Y., Cao, Y., Hu, H., Wei, Y., Zhang, Z., Lin, S., and Guo, B. (2021). Swin transformer: Hierarchical vision transformer using shifted windows. In Proceedings of the IEEE/CVF International Conference on Computer Vision, pp. 10012–10022.
27. He, K., Gan, C., Li, Z., Rekik, I., Yin, Z., Ji, W., and Gao, Y. (2022). Transformers in medical image analysis: A review. Preprint at arXiv.
28. Ghaffari Laleh, N., Truhn, D., Veldhuizen, G.P., Han, T., van Treeck, M., Buelow, R.D., Langer, R., Dislich, B., Boor, P., Schulz, V., and Kather, J.N. (2022). Adversarial attacks and adversarial robustness in computational pathology. Nat. Commun. *13*, 5711.
29. Wang, X., Yang, S., Zhang, J., Wang, M., Zhang, J., Yang, W., Huang, J., and Han, X. (2022). Transformer-based unsupervised contrastive learning for histopathological image classification. Med. Image Anal. *81*, 102559.
30. Chen, R.J., Chen, C., Li, Y., Chen, T.Y., Trister, A.D., Krishnan, R.G., and Mahmood, F. (2022). Scaling Vision Transformers to Gigapixel Images via Hierarchical Self-Supervised Learning. In Proceedings of the IEEE/CVF Conference on Computer Vision and Pattern Recognition, pp. 16144–16155.
31. Ghaffari Laleh, N., Muti, H.S., Loeffler, C.M.L., Echle, A., Saldanha, O.L., Mahmood, F., Lu, M.Y., Trautwein, C., Langer, R., Dislich, B., et al. (2022). Benchmarking weakly-supervised deep learning pipelines for whole slide classification in computational pathology. Med. Image Anal. *79*, 102474.
32. Shao, Z., Bian, H., Chen, Y., Wang, Y., Zhang, J., Ji, X., and Zhang, Y. (2021). TransMIL: Transformer based correlated multiple instance learning for whole slide image classification. In Advances in Neural Information Processing Systems, pp. 2136–2147.
33. Reisenbüchler, D., Wagner, S.J., Boxberg, M., and Peng, T. (2022). Local Attention Graph-based Transformer for Multi-target Genetic Alteration Prediction. Preprint at arXiv. [cs.CV].
34. Canny, J. (1986). A computational approach to edge detection. IEEE Trans. Pattern Anal. Mach. Intell. *8*, 679–698.
35. Wagner, S.J., Khalili, N., Sharma, R., Boxberg, M., Marr, C., de Back, W., and Peng, T. (2021). Structure-Preserving Multi-domain Stain Color Augmentation Using Style-Transfer with Disentangled Representations. In Medical Image Computing and Computer Assisted Intervention – MICCAI 2021 (Springer International Publishing)), pp. 257–266.
36. Kim, Y.J., Jang, H., Lee, K., Park, S., Min, S.-G., Hong, C., Park, J.H., Lee, K., Kim, J., Hong, W., et al. (2021). PAIP 2019: Liver cancer segmentation challenge. Med. Image Anal. *67*, 101854.
37. Edwards, N.J., Oberti, M., Thangudu, R.R., Cai, S., McGarvey, P.B., Jacob, S., Madhavan, S., and Ketchum, K.A. (2015). The CPTAC Data Portal: A Resource for Cancer Proteomics Research. J. Proteome Res. *14*, 2707–2713.
38. Vasaikar, S., Huang, C., Wang, X., Petyuk, V.A., Savage, S.R., Wen, B., Dou, Y., Zhang, Y., Shi, Z., Arshad, O.A., et al. (2019). Proteogenomic Analysis of Human Colon Cancer Reveals New Therapeutic Opportunities. Cell *177*, 1035–1049.e19.

39. Hoffmeister, M., Jansen, L., Rudolph, A., Toth, C., Kloor, M., Roth, W., Bläker, H., Chang-Claude, J., and Brenner, H. (2015). Statin use and survival after colorectal cancer: the importance of comprehensive confounder adjustment. J. Natl. Cancer Inst. *107*, djv045.

40. Brenner, H., Chang-Claude, J., Seiler, C.M., and Hoffmeister, M. (2011). Long-term risk of colorectal cancer after negative colonoscopy. J. Clin. Oncol. *29*, 3761–3767.

41. Grabsch, H., Dattani, M., Barker, L., Maughan, N., Maude, K., Hansen, O., Gabbert, H.E., Quirke, P., and Mueller, W. (2006). Expression of DNA double-strand break repair proteins ATM and BRCA1 predicts survival in colorectal cancer. Clin. Cancer Res. *12*, 1494–1500.

42. Gray, R.T., Loughrey, M.B., Bankhead, P., Cardwell, C.R., McQuaid, S., O'Neill, R.F., Arthur, K., Bingham, V., McGready, C., Gavin, A.T., et al. (2017). Statin use, candidate mevalonate pathway biomarkers, and colon cancer survival in a population-based cohort study. Br. J. Cancer *116*, 1652–1659.

43. Gray, R.T., Cantwell, M.M., Coleman, H.G., Loughrey, M.B., Bankhead, P., McQuaid, S., O'Neill, R.F., Arthur, K., Bingham, V., McGready, C., et al. (2017). Evaluation of PTGS2 Expression, PIK3CA Mutation, Aspirin Use and Colon Cancer Survival in a Population-Based Cohort Study. Clin. Transl. Gastroenterol. *8*, e91.

44. Morton, D., Seymour, M., Magill, L., Handley, K., Glasbey, J., Glimelius, B., Palmer, A., Seligmann, J., Laurberg, S., Murakami, K., et al. (2023). Preoperative Chemotherapy for Operable Colon Cancer: Mature Results of an International Randomized Controlled Trial. J. Clin. Oncol. *41*, 1541–1552.

45. Hawkins, N. (2011). MCO Study Tumour Collection.

46. Jonnagaddala, J., Croucher, J.L., Jue, T.R., Meagher, N.S., Caruso, L., Ward, R., and Hawkins, N.J. (2016). Integration and Analysis of Heterogeneous Colorectal Cancer Data for Translational Research. Stud. Health Technol. Inf. *225*, 387–391.

47. Ward, R., and Hawkins, N. Molecular and Cellular Oncology (MCO) Study Data. UNSW Australia. doi

48. (2015). MCO Study Whole Slide Image Collection.

49. Shulman, K., Barnett-Griness, O., Friedman, V., Greenson, J.K., Gruber, S.B., Lejbkowicz, F., and Rennert, G. (2018). Outcomes of Chemotherapy for Microsatellite Instable-High Metastatic Colorectal Cancers. JCO Precis. Oncol. *2*, 1–10.

50. van den Brandt, P.A., Goldbohm, R.A., van 't Veer, P., Volovics, A., Hermus, R.J., and Sturmans, F. (1990). A large-scale prospective cohort study on diet and cancer in The Netherlands. J. Clin. Epidemiol. *43*, 285–295.

51. Offermans, K., Jenniskens, J.C., Simons, C.C., Samarska, I., Fazzi, G.E., Smits, K.M., Schouten, L.J., Weijenberg, M.P., Grabsch, H.I., and van den Brandt, P.A. (2022). Expression of proteins associated with the Warburg-effect and survival in colorectal cancer. J. Pathol. Clin. Res. *8*, 169–180.

52. Quirke, P., and Morris, E. (2007). Reporting colorectal cancer. Histopathology *50*, 103–112.

53. Quasar Collaborative Group, Gray, R., Barnwell, J., McConkey, C., Hills, R.K., Williams, N.S., and Kerr, D.J. (2007). Adjuvant chemotherapy versus observation in patients with colorectal cancer: a randomised study. Lancet *370*, 2020–2029.

54. Cancer Genome Atlas Network (2012). Comprehensive molecular characterization of human colon and rectal cancer. Nature *487*, 330–337.

55. Isella, C., Cantini, L., Bellomo, S.E., and Medico, E. (2014). TCGA CRC 450 Dataset.

56. Iveson, T.J., Kerr, R.S., Saunders, M.P., Cassidy, J., Hollander, N.H., Tabernero, J., Haydon, A., Glimelius, B., Harkin, A., Allan, K., et al. (2018). 3 versus 6 months of adjuvant oxaliplatin-fluoropyrimidine combination therapy for colorectal cancer (SCOT): an international, randomised, phase 3, non-inferiority trial. Lancet Oncol. *19*, 562–578.

57. West, N.P., Gallop, N., Kaye, D., Glover, A., Young, C., Hutchins, G.G.A., Brockmoeller, S.F., Westwood, A.C., Rossington, H., and Quirke, P.; Yorkshire Cancer Research Bowel Cancer Improvement Programme Group (2021). Lynch syndrome screening in colorectal cancer: results of a prospective two-year regional programme validating the NICE diagnostics guidance pathway across a 5.2 million population. Histopathology *79*, 690–699.

58. Taylor, J., Wright, P., Rossington, H., Mara, J., Glover, A., West, N., Morris, E., and Quirke, P.; YCR BCIP study group (2019). Regional multidisciplinary team intervention programme to improve colorectal cancer outcomes: study protocol for the Yorkshire Cancer Research Bowel Cancer Improvement Programme (YCR BCIP). BMJ Open *9*, e030618.

59. Loshchilov, I., and Hutter, F. (2017). Decoupled Weight Decay Regularization. Preprint at arXiv. [cs.LG].

60. Kingma, D.P., and Ba, J. (2014). Adam: A Method for Stochastic Optimization. Preprint at arXiv. [cs.LG].

61. Smith, L.N., and Topin, N. (2019). Super-convergence: very fast training of neural networks using large learning rates. In Artificial Intelligence and Machine Learning for Multi-Domain Operations Applications (SPIE)), pp. 369–386.

62. Abnar, S., and Zuidema, W. (2020). Quantifying Attention Flow in Transformers. Preprint at arXiv. [cs.LG].

63. Fu, Y., Jung, A.W., Torne, R.V., Gonzalez, S., Vöhringer, H., Shmatko, A., Yates, L., Jimenez-Linan, M., Moore, L., and Gerstung, M. (2020). Pan-cancer Computational Histopathology Reveals Mutations, Tumor Composition and Prognosis.

64. Kather, J.N., Heij, L.R., Grabsch, H.I., Loeffler, C., Echle, A., Muti, H.S., Krause, J., Niehues, J.M., Sommer, K.A.J., Bankhead, P., et al. (2020). Pan-cancer image-based detection of clinically actionable genetic alterations. Nat. Can. (Ott.) *1*, 789–799.

65. Cercek, A., Lumish, M., Sinopoli, J., Weiss, J., Shia, J., Lamendola-Essel, M., El Dika, I.H., Segal, N., Shcherba, M., Sugarman, R., et al. (2022). PD-1 Blockade in Mismatch Repair–Deficient, Locally Advanced Rectal Cancer. N. Engl. J. Med. *386*, 2363–2376.

66. Hendricks, A., Amallraja, A., Meißner, T., Forster, P., Rosenstiel, P., Burmeister, G., Schafmayer, C., Franke, A., Hinz, S., Forster, M., and Williams, C.B. (2020). Stage IV Colorectal Cancer Patients with High Risk Mutation Profiles Survived 16 Months Longer with Individualized Therapies. Cancers *12*, 393.

67. Bera, K., Schalper, K.A., Rimm, D.L., Velcheti, V., and Madabhushi, A. (2019). Artificial intelligence in digital pathology - new tools for diagnosis and precision oncology. Nat. Rev. Clin. Oncol. *16*, 703–715.

68. Coudray, N., Ocampo, P.S., Sakellaropoulos, T., Narula, N., Snuderl, M., Fenyö, D., Moreira, A.L., Razavian, N., and Tsirigos, A. (2018). Classification and mutation prediction from non-small cell lung cancer histopathology images using deep learning. Nat. Med. *24*, 1559–1567.

69. Kleppe, A., Skrede, O.-J., De Raedt, S., Hveem, T.S., Askautrud, H.A., Jacobsen, J.E., Church, D.N., Nesbakken, A., Shepherd, N.A., Novelli, M., et al. (2022). A clinical decision support system optimising adjuvant chemotherapy for colorectal cancers by integrating deep learning and pathological staging markers: a development and validation study. Lancet Oncol. *23*, 1221–1232.

70. Campanella, G., Hanna, M.G., Geneslaw, L., Miraflor, A., Werneck Krauss Silva, V., Busam, K.J., Brogi, E., Reuter, V.E., Klimstra, D.S., and Fuchs, T.J. (2019). Clinical-grade computational pathology using weakly supervised deep learning on whole slide images. Nat. Med. *25*, 1301–1309.

71. National Institute for Health and Care Excellence (NICE) (2020). Molecular Testing Strategies for Lynch Syndrome in People with Colorectal Cancer (NICE Guidance). https://www.nice.org.uk/guidance/ng151.

72. Liu, Y., Sethi, N.S., Hinoue, T., Schneider, B.G., Cherniack, A.D., Sanchez-Vega, F., Seoane, J.A., Farshidfar, F., Bowlby, R., Islam, M., et al. (2018). Comparative Molecular Analysis of Gastrointestinal Adenocarcinomas. Cancer Cell *33*, 721–735.e8.

STAR★METHODS

KEY RESOURCES TABLE

REAGENT or RESOURCE	SOURCE	IDENTIFIER
Deposited data		
The Cancer Genome Archive (TCGA)	https://portal.gdc.cancer.gov/	RRID:SCR_003193
Clinical Proteomic Tumor Analysis Consortium (CPTAC)	https://proteomic.datacommons.cancer.gov/pdc/	RRID:SCR_017135
Software and algorithms		
Framework for experiments and model implementation	This manuscript; https://github.com/peng-lab/HistoBistro	https://zenodo.org/badge/latestdoi/613444008
The model has also been implemented in this framework	https://github.com/KatherLab/marugoto	

RESOURCE AVAILABILITY

Lead contact

Further information and requests regarding this manuscript should be sent to and will be fulfilled by the lead investigator, Jakob Nikolas Kather (jakob_nikolas.kather@tu-dresden.de).

Materials availability

We release all multi-cohort model weights created in this study under an open-source license. More specifically, the model for MSI high, *BRAF*, and *KRAS* detection.

Data and code availability

Some of the data that support the findings of this study are publicly available, and some are proprietary datasets provided under collaboration agreements. All data (including histological images) from the TCGA database are available at https://portal.gdc.cancer.gov/. All data from the CPTAC cohort are available at https://proteomic.datacommons.cancer.gov/. All molecular data for patients in the TCGA and CPTAC cohorts are available at https://cbioportal.org/. Data access for the Northern Ireland Biobank can be requested at http://www.nibiobank.org/for-researchers. Data access for the MCO cohort can be requested at https://researchdata.edu.au/mco-study-tumour-collection/1957427. All other data are under controlled access according to the local ethical guidelines and can only be requested directly from the respective study groups that independently manage data access for their study cohorts.

All code was implemented in Python using the DL framework PyTorch. All source codes to reproduce the experiments of this paper are available under an open-source license at https://github.com/peng-lab/HistoBistro (https://doi.org/10.5281/zenodo.8208791). The model is also implemented in the DL pipeline https://github.com/KatherLab/marugoto/tree/transformer. We release all model weights under an open-source license.

EXPERIMENTAL MODEL AND STUDY PARTICIPANT DETAILS

Ethics statement

In this study, we retrospectively analyzed anonymized patient samples from multiple academic institutions. At each of the following sites, the respective ethics board has given consent to this analysis: DACHS, Epi700, ERLANGEN, MAINZ, MECC, MUNICH, NLCS, QUASAR, FOxTROT, TRANSCOT, MCO. At the following sites, specific ethics approval was not required for a retrospective analysis of anonymized samples: CPTAC, DUSSEL, TCGA, GUANGZHOU, and YCR-BCIP. Our study adheres to STARD (Table S3).

Cohort description

Through coordination by the MSIDETECT consortium (www.msidetect.eu), we have collected over 20,000 H&E tissue sections of 13,689 patients with CRC from 16 patient cohorts in total, including two public databases (Figures 1D–1F). The cohorts obtained are as follows:

1. The public database “The Clinical Proteomic Tumor Analysis Consortium”, CPTAC (publicly available at https://pdc.cancer.gov/pdc/, USA)[37,38] which includes tumors of any stage;

2. DACHS (Darmkrebs: Chancen der Verhütung durch Screening, Southwest Germany),[39,40] a large population-based case-control and patient cohort study on CRC, including samples of patients with stages I-IV from different laboratories in southwestern Germany coordinated by the German Cancer Research Center (Heidelberg, Germany);
3. The DUSSEL (DUSSELdorf, Germany) cohort, a case series of CRC tumors resected with curative intent and collected at the Marien-Hospital in Duesseldorf, Germany, between January 1990 and December 1995[41];
4. Epi700 (Belfast, N. Ireland, UK),[42,43] a population-based cohort of stage II and III colon cancers treated by surgical resection between 2003 and 2008;
5. The ERLANGEN cohort, a CRC cohort collected at the Uniklinikum Erlangen in Germany between 2002 and 2010.
6. The "Fluoropyrimidine, Oxaliplatin, and Targeted Receptor pre-Operative Therapy for colon cancer cohort" (FOxTROT)[44] including pre-therapeutic biopsy and post-therapeutic resection tumors from UK sites;
7. The GUANGZHOU cohort, a small CRC case series of MSI-high cases collected in The Second Affiliated Hospital of Guangzhou Medical University, China;
8. The MAINZ cohort, a small CRC case series of biopsies collected in the University Medical Center Mainz in Germany.
9. The Molecular and Cellular Oncology Study (MCO) cohort[45–48] from the University of New South Wales, Australia;
10. MECC (Molecular Epidemiology of Colorectal Cancer, Israel),[49] a population-based case-control study in northern Israel;
11. The MUNICH (Munich, Germany) CRC series, a case series collected at the Technical University of Munich in Germany.
12. The NLCS (Netherlands Cohort Study, The Netherlands)[50,51] cohort, which contains tissue samples obtained from patients with any tumor stage as part of the Rainbow-TMA consortium study;
13. QUASAR, the "Quick and Simple and Reliable" trial investigating survival benefit of adjuvant chemotherapy in patients from the United Kingdom with mostly stage II tumors[52,53];
14. The public repository "The Cancer Genome Atlas", TCGA (publicly available at https://portal.gdc.cancer.gov/, USA)[54,55] which includes tumors of any stage;
15. The TransSCOT cohort, the translational arm of the SCOT trial, an "international, randomised, phase 3, non-inferiority trial" involving adult patients with high-risk stage II or stage III CRC[56];
16. The YCR-BCIP (Yorkshire Cancer Research Bowel Cancer Improvement Program, Yorkshire, United Kingdom [UK]), a population-based register of bowel cancer patients in Yorkshire, UK,[57,58] for which surgical resections and biopsies were available as separate cohorts.

Detailed clinicopathological variables are shown in Table S4. In all cohorts, formalin-fixed paraffin-embedded (FFPE) tissue was used. Slides have been scanned at their respective centers. For each patient, either an MSI status or an MMR status, obtained by PCR or IHC, respectively, is available. Although MSI status and MMR status are not fully concordant,[57] they are used interchangeably in clinical routine and grouped as a single category in this study. *KRAS* and *BRAF* mutational status are available for the cohorts DACHS, Epi700, NLCS, QUASAR, and TCGA.

METHOD DETAILS

Model description

Our biomarker prediction pipeline consists of three steps (Figure 1): i) the data pre-processing pipeline (Figure 1A), ii) the transformer-based feature extractor, and iii) the transformer-based aggregation module that yields the final prediction from the embeddings of all patches of a whole-slide image (WSI) (Figure 1B).

In the pre-processing pipeline, tissue regions are segmented using RGB thresholding and Canny edge detection[34] to detect background and blurry regions. We include all tiles from a WSI, i.e., both tumor and healthy tissue tiles, thus reducing the burden of manual annotations when applying the algorithm. Subsequently, the WSI is tessellated into tiles of size 512 × 512 pixels at 20× magnification with a resolution of 0.5 microns per pixel. To reduce the impact of the staining color on the model generalization, the tiles are stain-color augmented using a structure-preserving GAN trained on TCGA.[35]

We extract feature representations of dimension 768 for every tile using the CTransPath model.[29] (Figure 1B). The model architecture is based on a Swin Transformer[26] that combines the hierarchical structure of CNNs with the global self-attention modules of transformers by computing self-attention in a sliding-window fashion. Similar to CNNs, these are stacked to increase the receptive field in every stage. CTransPath consists of three convolutional layers at the beginning to facilitate local feature extraction and improve training stability,[29] followed by four Swin Transformer stages. Wang et al. trained the network using an unsupervised contrastive loss on data from TCGA and PAIP[36] from multiple organs and provided the weights for public use. The embeddings for each tile are stored for the subsequent training procedure.

The final part of the model takes all patches of a WSI as input and predicts one biomarker for all input patches in a weakly supervised manner (Figure 1B). Common attention-based MIL approaches[23] use a small neural network, which mostly consists of two layers, to compute patch importance based on the embeddings. Each weight is computed based on one patch and finally, all weights are normalized over the input elements. In contrast to this, in our model, the patch embeddings are passed into a transformer network using multi-headed self-attention that considers the patch embeddings as a sequence and relates each element to every other element. In particular, assuming that $x \in R^{n \times d}$ is the input sequence representing a WSI with n patch embeddings of dimension d, the self-attention layer computes a query-key product in the following way

$$SA(Q,K,V) = softmax\left(\frac{QK^T}{\sqrt{d_k}}\right)V,$$

where queries $Q \in \mathbb{R}^{n \times d_k}$, keys $K \in \mathbb{R}^{n \times d_k}$, and values $V \in \mathbb{R}^{n \times d_v}$.These are computed from the input sequence x by

$$Q = W_Q \cdot x, K = W_K \cdot x \text{ and } V = W_V \cdot x,$$

where $W_Q \in \mathbb{R}^{d \times d_k}$, $W_K \in \mathbb{R}^{d \times d_k}$, and $W_V \in \mathbb{R}^{d \times d_v}$ are learnable parameters. Multi-headed self-attention applies self-attention in every head and concatenates the heads in a weighted manner:

$$MSA(x) = concat(head_1, ..., head_h) \cdot W_O$$

where $head_i = SA(Q^{(i)}, K^{(i)}, V^{(i)})$ for $i \in \{1, ..., h\}$

and $W_O \in \mathbb{R}^{hd_v \times d}$ is learnable. We choose a small transformer network architecture consisting of two layers with each eight heads ($h = 8$), a latent dimension of 512, and the same dimension for query, keys, and values. Therefore, the latent dimension of each head is $d_v = d_k = 64$, such that $hd_v = 8 \cdot 64 = 512$.

Assuming that n is the number of patches per WSI, the embeddings of each patch $i \in \{1, \ldots, n\}$ are stacked to a sequence of dimension $n \times 768$ and are passed through a linear projection layer followed by the non-linear activation ReLU to reduce the dimension from 768 to 512. Subsequently, a class token is concatenated to the input, similar to the usage in vision transformers,[25] yielding an input of dimension $(n + 1) \times 512$ that is passed to the transformer layer. In each transformer layer, a block of layer normalization and multi-headed self-attention is followed by a block of layer normalization and a multi-layer perceptron (MLP), with skip connections applied across each block (Figure 1C).

After the two transformer layers, the class token of size 1×512 is passed into an MLP head. Depending on the number of class tokens used, this enables single-target or multi-target binary prediction. Instead of attaching a class token, all n sequence elements could be averaged to a single sequence element of size 1×512 and passed into the MLP head. The averaging approach achieves similar performance to the class token version (Table S2), but we decided to use the class token for better interpretability of the attention heads. We also compared our model architecture to the existing transformer-based aggregation module TransMIL[32] (Table S2).

Experimental setup and implementation details

We performed all experiments using 5-fold cross-validation with in-domain validation and testing. In this cross-validation variant, in-domain validation and test set are split off the full dataset on patient level, leaving 3-folds for training. By also cycling the in-domain test set through the complete dataset, we evaluated our model on more representative test sets than when fixing one smaller set for the dataset. During training, the validation set was used to determine the best model, which was finally evaluated on the test set. We further evaluated our models on external cohorts outside the dataset for out-of-domain testing.

The transformer models were trained with the AdamW[59] optimizer using weight decay and learning rate both of $2 * 10^{-5}$. All models were trained for eight epochs with batch size of one for two reasons: first, the sequences of embeddings had different lengths due to the variable number of tiles per WSI and could thus not be stacked to mini-batches of equal length inputs; second, limits in GPU capacity (32GB) because of the quadratic complexity of the self-attention mechanism and the large number of tiles per slide (up to 12,000, Figure S1). To account for the varying cohort size, we evaluated the models every 500 iterations for runs on single cohorts, and every 1000 iterations for runs on multiple cohorts.

For comparison, we implemented the attention-based MIL approach from Ilse et al.,[23] referred to as AttentionMIL. It provided the best results with Adam[60] optimizer, $1 * 10^{-2}$ as weight decay value, along with the fit-one-cycle learning rate scheduling policy[61] with a maximum learning rate of $1 * 10^{-4}$, trained over 32 epochs, and the first 25% of the cycle with increasing learning rate.

Visualization and explainability

The final prediction is retrieved via the class token that is attached to the input sequence. To visualize the contribution of each input patch, we employed attention rollout as introduced by Abnar and Zuidema.[62] To obtain the attention at the class token in the final layer, the attention maps of the preceding layers are multiplied recursively. Attention rollout thus quantifies to which extent each patch contributes to the final prediction in the class score. Additionally, we visualized the attention scores for each head in the transformer by taking the class token's self-attention, i.e., the query and key product. All presented attention scores were normalized to the range $[0, 1]$ and clamped to the lower and higher 5%-quantiles, respectively, for better visual interpretability.

To visualize whether a patch contributed toward a positive or negative classification outcome, we fed the patches one-by-one through the transformer and visualized the resulting classification scores of the model. These scores were naturally in the range $[0, 1]$ and can thus be directly visualized without further normalizing or clamping of values.

QUANTIFICATION AND STATISTICAL ANALYSIS

We used the area under the receiver operator curve (AUROC) as our main evaluation metric. Since our data are naturally highly imbalanced with respect to the target variables MSI, *BRAF*, and *KRAS* (Figures 1D and 1E), we further used the area under the

precision-recall curve (AUPRC) as a metric as this metric accounts better for class imbalances than the AUROC metric. The precision-recall curve relates the recall or specificity, i.e., the ratio of correctly positive predicted samples to all positive samples, to the precision, i.e., the ratio of correctly positive predicted samples to all positive predicted samples. For every experiment, we reported the mean and the standard deviation of respective 5-fold cross-validation's model in-domain and external test performances. We split the dataset into patient-wise training, validation, and internal test sets stratified by the target label, thus ensuring that every patient can only occur in one of these sets. The external test sets always consisted of different cohorts to better quantify the generalization properties of our algorithms.

Cancer Cell, Volume *41*

Supplemental information

Transformer-based biomarker prediction

from colorectal cancer

histology: A large-scale multicentric study

Sophia J. Wagner, Daniel Reisenbüchler, Nicholas P. West, Jan Moritz Niehues, Jiefu Zhu, Sebastian Foersch, Gregory Patrick Veldhuizen, Philip Quirke, Heike I. Grabsch, Piet A. van den Brandt, Gordon G.A. Hutchins, Susan D. Richman, Tanwei Yuan, Rupert Langer, Josien C.A. Jenniskens, Kelly Offermans, Wolfram Mueller, Richard Gray, Stephen B. Gruber, Joel K. Greenson, Gad Rennert, Joseph D. Bonner, Daniel Schmolze, Jitendra Jonnagaddala, Nicholas J. Hawkins, Robyn L. Ward, Dion Morton, Matthew Seymour, Laura Magill, Marta Nowak, Jennifer Hay, Viktor H. Koelzer, David N. Church, TransSCOT consortium, Christian Matek, Carol Geppert, Chaolong Peng, Cheng Zhi, Xiaoming Ouyang, Jacqueline A. James, Maurice B. Loughrey, Manuel Salto-Tellez, Hermann Brenner, Michael Hoffmeister, Daniel Truhn, Julia A. Schnabel, Melanie Boxberg, Tingying Peng, and Jakob Nikolas Kather

Supplementary Figures

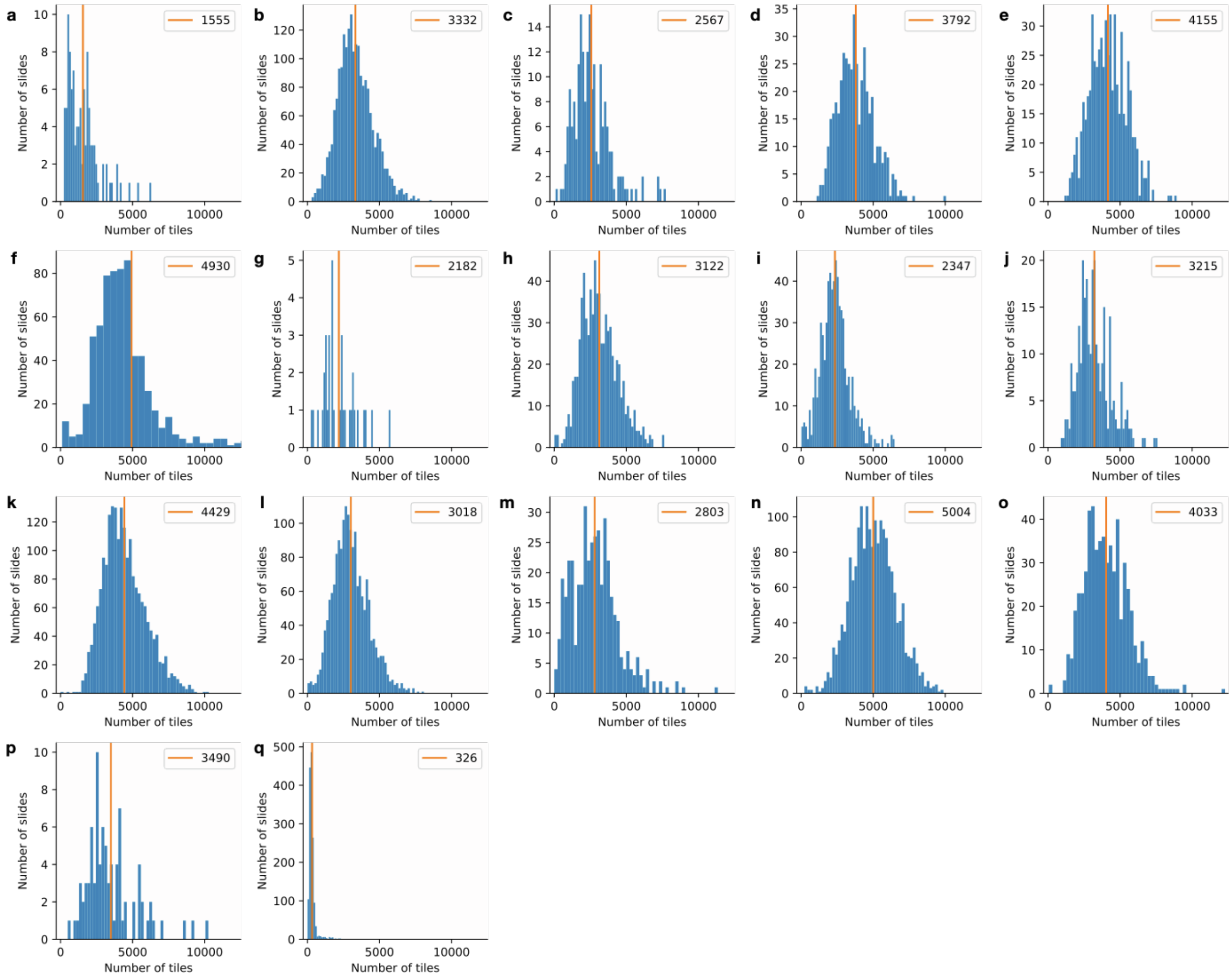

Supplementary Figure 1: **Distribution of the number of tiles per slide for all cohorts**, related to cohort overview in **Figure 1.** The mean of each distribution is highlighted in orange. a-o) Histogram of the distribution for all 15 cohorts of resections a) CPTAC, b) DACHS, c) DUSSEL, d) ERLANGEN, e) Epi700, f) FOXTROT, g) GUANGZHOU, h) MCO, i) MECC, j) MUNICH, k) NLCS, l) QUASAR, m) TCGA, n) TRANSCOT, and o) YCR-BCIP. p-q) Histogram of the distribution for the biopsy cohorts p) YCR-BCIP and q) MAINZ.

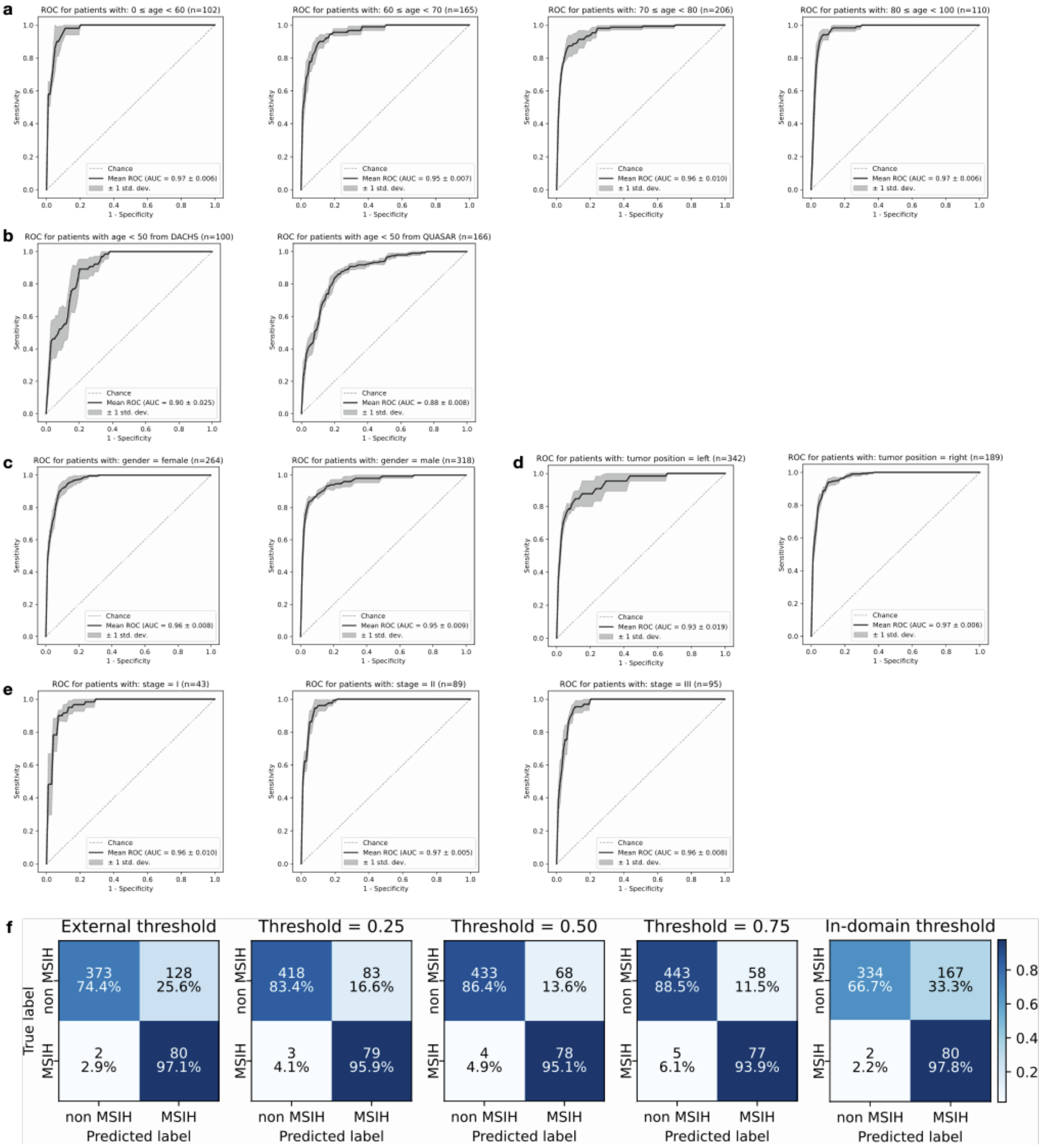

Supplementary Figure 2: **Analysis of clinico-pathological features with receiver operator curves (a-e) and confusion matrix for different classification thresholds (f)**, related to results in **Figure 2**. a) Age groups, below 60, between 60 and 70, between 70 and 80, and above 80 for multi-cohort model evaluated on YCR-BCIP. b) Early onset cancer: model trained on QUASAR, tested on DACHS with 100 patients younger than 50 years and model trained on DACHS, tested on QUASAR with 166 patients younger than 50 years. Both cohorts were chosen because they contain a sufficient number of patients under 50 in contrast to the other cohorts in this study. c-d) Multi-cohort model evaluated on YCR-BCIP. c) ROCs of female and male patients. d) ROCs for patiets with left- vs. right-sided tumors. d) ROCs for patients with tumors in stage I, II, and III. f) First column shows the threshold determined on the external tests, such that 0.95 sensitivity is reached. Second to forth column show fixed thresholds (0.25, 0.5, 0.75, respectively). Last column shows the threshold determined on the in-domain test set, such that 0.95 sensitivity is reached. The results show the average of the model trained on the large multi-centric cohort DACHS, NLCS, QUASAR, and TCGA across all five folds. Results of multi-cohort model evaluated on YCR-BCIP.

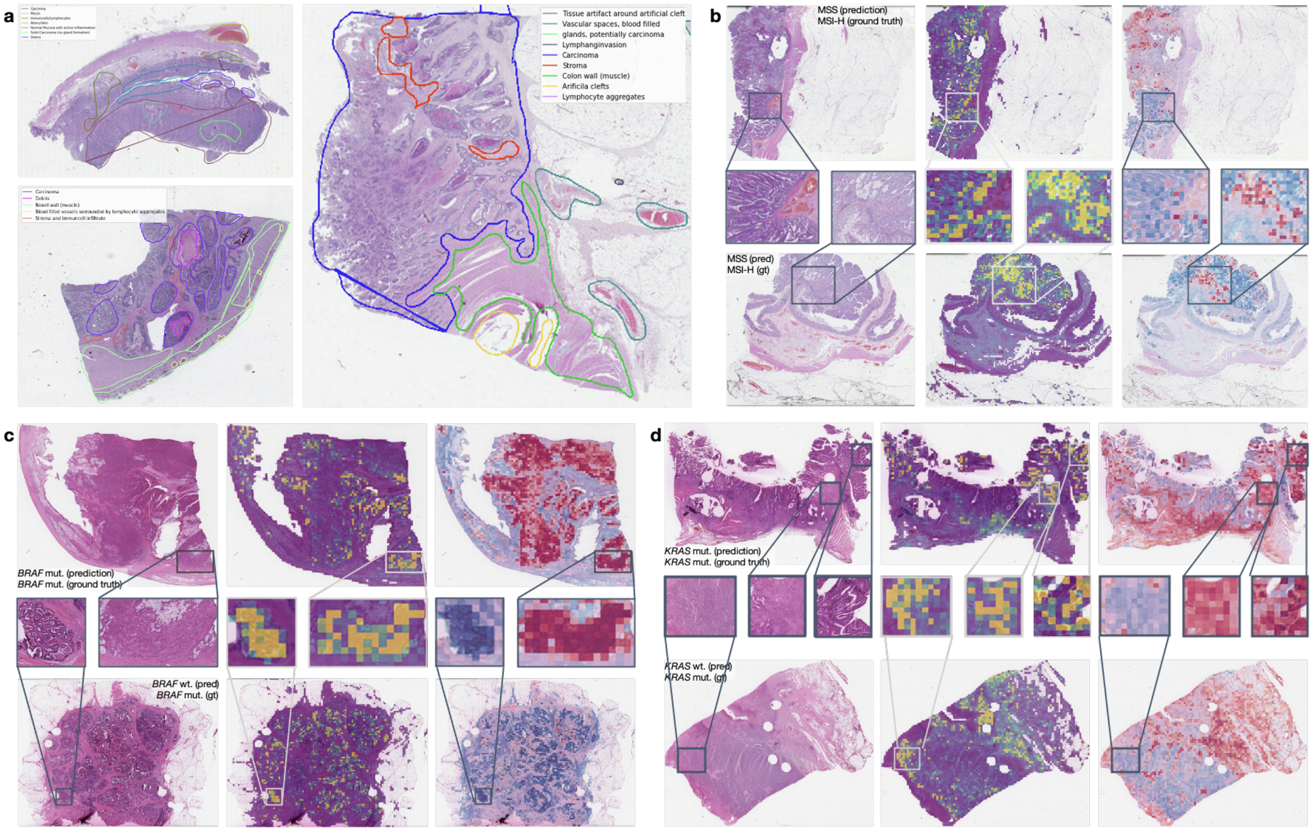

Supplementary Figure 3: **Annotations an additional cases for the interpretability analysis in Figure 3.** a) Manual annotation by a pathologist of the three test cases used for attention visualization from the YCR-BCIP cohort. b-d) Attention and classification score visualizations: left) original WSIs, center) attention score map, right) patch-wise classification score map. b) False negative cases in YCR-BCIP cohort for model trained on multi-cohort dataset. c) Model trained on the cohorts DACHS, QUASAR, MCO, NLCS, TCGA, for *BRAF* predictions, samples from the test cohort Epi700. d) Model trained on the cohorts DACHS, QUASAR, MCO, NLCS, TCGA, for *KRAS* predictions, samples from the test cohort Epi700.

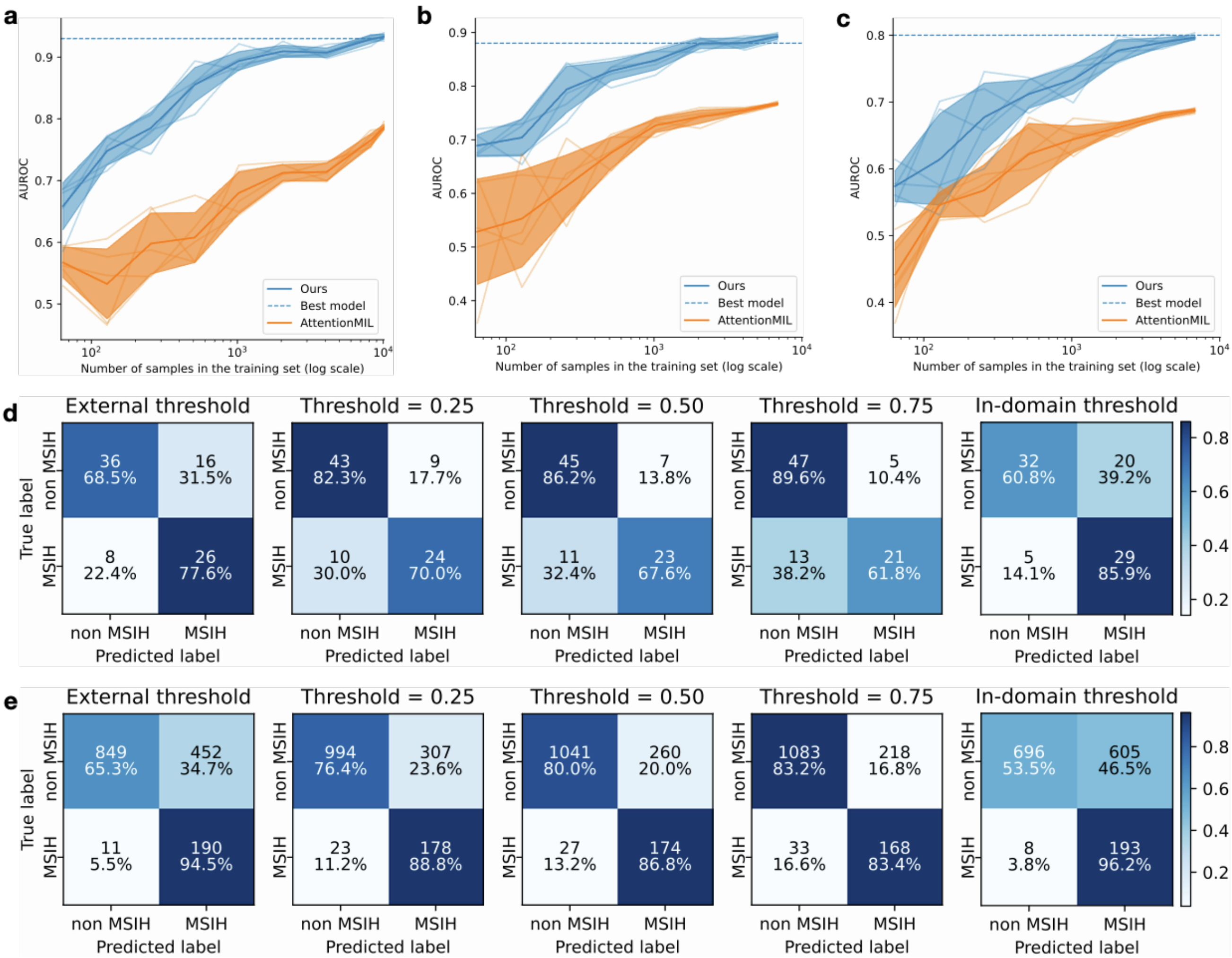

Supplementary Figure 4: Data efficiency analysis and confusion matrices, related to **Figure 4**. a-c) AUROC scores depending on the number of patients available for training. The samples were randomly drawn from all resection cohorts with available labels except the external test cohort. a) MSI prediction on YCR-BCIP. b) *BRAF* prediction on Epi700. c) *KRAS* prediction on Epi700. d-e) First column shows the threshold determined on the external tests, such that 0.95 sensitivity is reached. Second to forth column show fixed thresholds (0.25, 0.5, 0.75, respectively). Last column shows the threshold determined on the in-domain test set, such that 0.95 sensitivity is reached. d) Results of multi-cohort model evaluated on the biopsy cohort MAINZ. e) Results of multi-cohort model evaluated on the biopsy cohort YCR-BCIP.

Supplementary Tables

Suppl. Table 1: Multi-cohort experiments with statistical endpoints, related to **Figure 2.** Multi-cohort dataset consisting of CPTAC, DACHS, DUSSEL, Epi700, ERLANGEN, FOxTROT, MCO, MECC, MUNICH, QUASAR, RAINBOW, TCGA, TRANSCOT (all resection cohorts except YCR-BCIP and GUANGZHOU). The models were trained with HistAuGAN stain color augmentation, CTransPath as feature extractor and our transformer model with class token as aggregation model. The thresholds 0.9, 0.925, and 0.95 were determined on the in-domain test set and used for the evaluation on the external test sets. All results for sensitivity, negative predictive value (NPV), and sensitivity are averaged over the five folds.

Train	Test	Target	AUROC mean	AUROC std dev	Sensitivity (0.95)	NPV (0.95)	Specificity (0.95)	Sensitivity (0.925)	NPV (0.925)	Specificity (0.925)	Sensitivity (0.9)	NPV (0.9)	Specificity (0.9)
Multi-cohort dataset	Multi-cohort dataset	MSI high	0.93	0.0084	0.95	0.99	0.61	0.92	0.98	0.72	0.9	0.98	0.78
Multi-cohort dataset	YCR-BCIP-resections	MSI high	0.97	0.0041	0.995	0.998	0.41	0.99	0.995	0.56	0.98	0.995	0.67
Multi-cohort dataset	GUANGZHOU	MSI high	-	-	0.92	-	-	0.9	-	-	0.86	-	-
Multi-cohort dataset	YCR-BCIP-biopsies	MSI high	0.92	0.0066	0.99	0.995	0.31	0.98	0.99	0.44	0.96	0.99	0.54
Multi-cohort dataset	MAINZ	MSI high	0.86	0.0174	0.93	0.9	0.39	0.91	0.9	0.51	0.86	0.88	0.61
DACHS, QUASAR, NLCS, TCGA, MCO	DACHS, QUASAR, NLCS, TCGA, MCO	*BRAF*	0.88	0.0127	0.95	0.99	0.49	0.92	0.99	0.61	0.9	0.98	0.68
DACHS, QUASAR, NLCS, TCGA, MCO	Epi700	*BRAF*	0.88	0.0103	0.94	0.98	0.55	0.91	0.98	0.65	0.88	0.97	0.71
DACHS, QUASAR, NLCS, TCGA, MCO	DACHS, QUASAR, NLCS, TCGA, MCO	*KRAS*	0.71	0.0053	0.95	0.87	0.18	0.93	0.93	0.23	0.9	0.84	0.29
DACHS, QUASAR, NLCS, TCGA, MCO	Epi700	*KRAS*	0.80	0.0124	0.98	0.95	0.16	0.98	0.93	0.21	0.97	0.93	0.28

Suppl. Table 2: Ablation study on architecture choices, related to **Figure 2**. The models were trained with the same pre-processing and feature extractor, only varying the architecture of the aggregation model. All models were trained with 5-fold cross validation.

**Number**	**Train**	**Test**	**Target**	**Normali-zation**	**Feature Extraction**	**Aggregation Model**	**AUROC mean**	**AUROC std dev**	**AUPRC mean**	**AUPRC std dev**	**F1 (0.5) mean**	**F1 (0.5) std dev**	**F1 (gmean) mean**	**F1 (gmean) std dev**
2.1.1	DACHS, NLCS, QUASAR, TCGA	DACHS, NLCS, QUASAR, TCGA	MSI-H	Macenko	CTransPath[29]	Transformer with class token (ours)	0.95	0.0078	0.74	0.0284	0.83	0.1257	0.80	0.1370
2.2.1	DACHS, NLCS, QUASAR, TCGA	YCR-BCIP	MSI-H	Macenko	CTransPath[29]	Transformer with class token (ours)	**0.97**	0.0041	0.83	0.0266	**0.83**	0.1145	**0.84**	0.1108
2.3.1	DACHS, NLCS, QUASAR, TCGA	YCR-BCIP-biopsies	MSI-H	Macenko	CTransPath[29]	Transformer with class token (ours)	**0.91**	0.0094	0.63	0.0149	**0.77**	0.1616	**0.74**	0.1622
2.1.2	DACHS, NLCS, QUASAR, TCGA	DACHS, NLCS, QUASAR, TCGA	MSI-H	Macenko	CTransPath[29]	Transformer with global averaging (ours)	0.95	0.0091	0.76	0.0224	0.83	0.1268	0.81	0.1322
2.2.2	DACHS, NLCS, QUASAR, TCGA	YCR-BCIP	MSI-H	Macenko	CTransPath[29]	Transformer with global averaging (ours)	**0.97**	0.0042	**0.84**	0.0131	0.82	0.1272	0.83	0.1191
2.3.2	DACHS, NLCS, QUASAR, TCGA	YCR-BCIP-biopsies	MSI-H	Macenko	CTransPath[29]	Transformer with global averaging (ours)	**0.91**	0.0078	**0.64**	0.0206	0.75	0.1795	**0.74**	0.1582
2.1.3	DACHS, NLCS, QUASAR, TCGA	DACHS, NLCS, QUASAR, TCGA	MSI-H	Macenko	CTransPath[29]	AttentionMIL[23]	0.94	0.0103	0.71	0.0184	0.79	0.1477	0.77	0.1543
2.2.3	DACHS, NLCS, QUASAR, TCGA	YCR-BCIP	MSI-H	Macenko	CTransPath[29]	AttentionMIL[23]	0.96	0.0025	0.80	0.0101	0.78	0.1413	0.82	0.1202
2.3.3	DACHS, NLCS, QUASAR, TCGA	YCR-BCIP-biopsies	MSI-H	Macenko	CTransPath[29]	AttentionMIL[23]	0.90	0.0042	0.60	0.0154	0.76	0.1601	**0.74**	0.1595
2.1.4	DACHS, NLCS, QUASAR, TCGA	DACHS, NLCS, QUASAR, TCGA	MSI-H	Macenko	CTransPath[29]	TransMIL[32]	0.94	0.0101	0.72	0.0379	0.82	0.1387	0.79	0.1500
2.2.4	DACHS, NLCS, QUASAR, TCGA	YCR-BCIP	MSI-H	Macenko	CTransPath[29]	TransMIL[32]	0.96	0.0033	0.79	0.0200	0.84	0.1157	0.83	0.1155
2.3.4	DACHS, NLCS, QUASAR, TCGA	YCR-BCIP-biopsies	MSI-H	Macenko	CTransPath[29]	TransMIL[32]	0.89	0.0122	0.57	0.0178	0.72	0.2215	0.70	0.1736

Suppl. Table 3: STARD (STAndards for the Reporting of Diagnostic accuracy studies) Checklist, related to STAR Methods**.**

**Section & Topic**	**No**	**Item**	**Reported**
TITLE OR ABSTRACT	1	Identification as a study of diagnostic accuracy using at least one measure of accuracy (such as sensitivity, specificity, predictive values, or AUC)	yes
ABSTRACT	2	Structured summary of study design, methods, results, and conclusions (for specific guidance, see STARD for Abstracts)	yes
INTRODUCTION	3	Scientific and clinical background, including the intended use and clinical role of the index test	yes
	4	Study objectives and hypotheses	yes
METHODS<br>Study design	5	Whether data collection was planned before the index test and reference standard were performed (prospective study) or after (retrospective study)	
METHODS<br>Participants	6	Eligibility criteria	
	7	On what basis potentially eligible participants were identified (such as symptoms, results from previous tests, inclusion in registry)	
	8	Where and when potentially eligible participants were identified (setting, location and dates)	
	9	Whether participants formed a consecutive, random or convenience series	
METHODS<br>Test methods	10a	Index test, in sufficient detail to allow replication	yes
	10b	Reference standard, in sufficient detail to allow replication	yes
	11	Rationale for choosing the reference standard (if alternatives exist)	
	12a	Definition of and rationale for test positivity cut-offs or result categories of the index test, distinguishing pre-specified from exploratory	yes
	12b	Definition of and rationale for test positivity cut-offs or result categories of the reference standard, distinguishing pre-specified from exploratory	
	13a	Whether clinical information and reference standard results were available to the performers/readers of the index test	
	13b	Whether clinical information and index test results were available to the assessors of the reference standard	
METHODS<br>Analysis	14	Methods for estimating or comparing measures of diagnostic accuracy	yes
	15	How indeterminate index test or reference standard results were handled	
	16	How missing data on the index test and reference standard were handled	yes
	17	Any analyses of variability in diagnostic accuracy, distinguishing pre-specified from exploratory	
	18	Intended sample size and how it was determined	yes
RESULTS<br>Participants	19	Flow of participants, using a diagram	

Section & Topic	No	Item	Reported
	20	Baseline demographic and clinical characteristics of participants	yes
	21a	Distribution of severity of disease in those with the target condition	
	21b	Distribution of alternative diagnoses in those without the target condition	yes
	22	Time interval and any clinical interventions between index test and reference standard	
RESULTS<br>Test results	23	Cross tabulation of the index test results (or their distribution) by the results of the reference standard	
	24	Estimates of diagnostic accuracy and their precision (such as 95% confidence intervals)	yes
	25	Any adverse events from performing the index test or the reference standard	
DISCUSSION	26	Study limitations, including sources of potential bias, statistical uncertainty, and generalisability	yes
	27	Implications for practice, including the intended use and clinical role of the index test	
OTHER<br>INFORMATION	28	Registration number and name of registry	
	29	Where the full study protocol can be accessed	
	30	Sources of funding and other support; role of funders	yes

Suppl. Table 4: Patient cohorts used in this study and their characteristics, related to Figure 1. Clinico-pathological data were provided by the respective study principal investigators. In all cases, the TNM version from the original study registry was used. Information about the localization of the tumor was either provided as a binary variable (left-sided vs. right-sided) by the study site or assigned by the authors as follows: the cecum, ascending colon, hepatic flexure and transverse colon were defined as a right-sided tumor location whereas the splenic flexure, descending colon, sigmoid colon and rectum were defined as left-sided. *Number of patients before dropout of samples. **for the MECC cohort, these statistics refer to the cases with available MSI/dMMR status only.

	CPTAC	DACHS	DUSSEL	Epi700	ERLANGEN	FOxTROT	GUANGZHOU	MAINZ	MCO	MECC**	MUNICH	NLCS	QUASAR	TCGA	TRANSCOT	YCR-BCIP	YCR-BCIP biopsies
**Origin**	United States	Germany	Germany	Northern Ireland	Germany	United Kingdom	China	Germany	Australia	Israel	Germany	Nether-lands	United Kingdom	United States	United Kingdom	United Kingdom	United Kingdom
**Number of patients***	110	2448	330	661	627	1053	35	90	1511	683	292	2452	2190	632	1988	889	1557
**WSI format**	SVS	SVS	SVS	SVS	MRXS	SVS	MRXS	SVS	SVS	TIF	SVS	TIFF/SVS	SVS	SVS	SVS	SVS	SVS
**MSI-H/dMMR ground truth**	MuTect2[38]	PCR 3-plex	IHC 2-plex	PCR/IHC consensus	IHC 4-plex	IHC 4-plex	IHC 4-plex	IHC 4-plex	IHC 4-plex	PCR 5-plex	IHC 4-plex	IHC 2-plex	IHC 4-plex / IHC 2-plex	PCR 5-plex[72]	IHC	IHC 4-plex	IHC 4-plex
**MSI-H/dMMR, n (%)**	24 (22%)	210 (9%)	45 (14%)	134 (20%)	113 (18%)	185 (18%)	35 (100%)	36 (40%)	238 (16%)	106 (16%)	34 (12%)	259 (11%)	246 (11%)	65 (10%)	229 (12%)	129 (15%)	211 (14%)
**MSS/pMMR, n (%)**	81 (74%)	1836 (75%)	268 (81%)	469 (71%)	407 (65%)	728 (69%)	0 (0%)	54 (60%)	1268 (85%)	577 (84%)	258 (88%)	2193 (89%)	1529 (70%)	392 (62%)	1759 (88%)	760 (85%)	1346 (86%)
**Mean age at diagnosis (std. dev.)**	65.67 (11.38)	68.46 (10.82)	68.57 (11.77)	70.63 (11.4)	N/A	N/A	N/A	N/A	68.4 (12.51)	69.8	56.1 (11.84)	73.71 (6.04)	62.20 (9.60)	66.42 (12.67)	63.84 (9.11)	70.31 (9.97)	71.79 (9.97)
**Colon cancer, n (%)**	110 (100%)	1488 (61%)	204 (62%)	659 (99.7%)	N/A	N/A	N/A	N/A	955 (63%)	530 (78%)	N/A	1730 (71%)	1474 (67%)	341 (54%)	N/A	667 (75%)	876 (56%)
**Rectal cancer, n (%)**	0 (0%)	960 (39%)	116 (35%)	2 (0.3%)	N/A	N/A	N/A	N/A	552 (37%)	123 (18%)	N/A	722 (29%)	526 (24%)	118 (19%)	N/A	215 (24%)	662 (43%)
**Site unknown, n (%)**	0 (0%)	0 (0%)	10 (3%)	0 (0%)	N/A	N/A	N/A	N/A	4 (0%)	30 (4%)	N/A	0 (0%)	190 (9%)	173 (27%)	N/A	7 (1%)	19 (1%)
**Female, n (%)**	65 (59%)	1012 (41%)	181 (55%)	303 (46%)	N/A	N/A	N/A	N/A	685 (45%)	320 (47%)	132 (45%)	1079 (44%)	848 (39%)	292 (46%)	802 (40%)	395 (44%)	620 (40%)
**Male, n (%)**	45 (41%)	1436 (59%)	149 (45%)	358 (54%)	N/A	N/A	N/A	N/A	826 (55%)	363 (53%)	160 (55%)	1373 (56%)	1334 (61%)	322 (51%)	1186 (60%)	494 (56%)	933 (60%)
**gender unknown**	0 (0%)	0 (0%)	0 (0%)	0 (0%)	N/A	N/A	N/A	N/A	0 (0%)	0 (0%)	0 (0%)	0 (0%)	8 (0%)	18 (3%)	0 (0%)	0 (0%)	4 (0%)
**UICC stage I, n (%)**	12 (11%)	485 (20%)	76 (23%)	0 (0%)	N/A	N/A	N/A	N/A	289 (19%)	94 (14%)	52 (18%)	485 (20%)	1 (0%)	76 (12%)	N/A	169 (19%)	2 (0%)
**UICC stage II, n (%)**	42 (38%)	801 (33%)	138 (42%)	394 (60%)	N/A	N/A	N/A	N/A	542 (36%)	335 (49%)	118 (40%)	918 (37%)	1988 (91%)	166 (26%)	N/A	317 (36%)	2 (0%)
**UICC stage III, n (%)**	48 (44%)	822 (34%)	110 (33%)	267 (40%)	N/A	N/A	N/A	N/A	503 (33%)	123 (18%)	82 (28%)	641 (26%)	192 (9%)	140 (22%)	N/A	370 (42%)	5 (0%)
**UICC stage IV, n**	8	337	6	0	N/A	N/A	N/A	N/A	177	67	39	341	0	63	N/A	0	0

	CPTAC	DACHS	DUSSEL	Epi700	ERLANGEN	FOxTROT	GUANGZHOU	MAINZ	MCO	MECC**	MUNICH	NLCS	QUASAR	TCGA	TRANSCOT	YCR-BCIP	YCR-BCIP biopsies
**(%)**	(7%)	(14%)	(2%)	(0%)					(12%)	(10%)	(13%)	(14%)	(0%)	(10%)		(0%)	(0%)
**UICC stage unknown, n (%)**	0 (0%)	3 (0%)	0 (0%)	0 (0%)	N/A	N/A	N/A	N/A	0 (0%)	64 (9%)	1 (0%)	67 (3%)	9 (0%)	187 (30%)	N/A	33 (3%)	1548 (99%)
***BRAF* mutation, n (%)**	N/A	151 (6%)	N/A	91 (14%)	N/A	N/A	N/A	N/A	190 (13%)	49 (7%)	N/A	305 (12%)	120 (5%)	63 (10%)	N/A	75 (8%)	139 (9%)
***BRAF* wild type, n (%)**	N/A	1930 (79%)	N/A	550 (84%)	N/A	N/A	N/A	N/A	1271 (84%)	570 (83%)	N/A	1733 (71%)	1358 (62%)	471 (75%)	N/A	32 (4%)	36 (2%)
***BRAF* status unknown, n (%)**	N/A	367 (15%)	N/A	16 (2%)	N/A	N/A	N/A	N/A	50 (3%)	64 (9%)	N/A	414 (17%)	712 (33%)	98 (15%)	N/A	782 (88%)	1382 (89%)
***KRAS* mutation, n (%)**	N/A	677 (28%)	N/A	247 (38%)	N/A	N/A	N/A	N/A	460 (30%)	252 (37%)	N/A	698 (28%)	555 (25%)	218 (34%)	N/A	N/A	N/A
***KRAS* wild type, n (%)**	N/A	1397 (57%)	N/A	398 (61%)	N/A	N/A	N/A	N/A	1001 (66%)	405 (59%)	N/A	1335 (54%)	882 (40%)	316 (50%)	N/A	N/A	N/A
***KRAS* status unknown**	N/A	347 (15%)	N/A	12 (2%)	N/A	N/A	N/A	N/A	50 (3%)	26 (4%)	N/A	419 (17%)	753 (35%)	98 (16%)	N/A	N/A	N/A
**right-sided tumor, n (%)**	58 (53%)	819 (33%)	72 (22%)	375 (57%)	N/A	N/A	N/A	N/A	589 (39%)	238 (35%)	53 (18%)	946 (39%)	754 (34%)	176 (28%)	779 (39%)	331 (37%)	395 (25%)
**left-sided tumor, n (%)**	51 (46%)	1607 (66%)	226 (68%)	280 (42%)	N/A	N/A	N/A	N/A	918 (61%)	409 (60%)	239 (82%)	1506 (61%)	1158 (53%)	248 (39%)	1180 (60%)	486 (55%)	1055 (68%)
**sidedness unknown, n (%)**	1 (1%)	22 (1%)	32 (10%)	6 (1%)	N/A	N/A	N/A	N/A	4 (0%)	36 (5%)	0 (0%)	0 (0%)	150 (13%)	208 (33%)	29 (1%)	72 (8%)	107 (7%)

Suppl. Table 5 Mean AUROC scores of 5-fold CV training on single cohorts, evaluated on all other cohorts, related to **Figure 2**. The training cohorts are listed in the columns and entries in the diagonal are in-domain test results.

Train (↓)	**NLCS**	**DACHS**	**TRANSCOT**	**QUASAR**	**MCO**	**YCR-BCIP**	**FOxTROT**	**MECC**	**Epi700**	**ERLANGEN**	**TCGA**	**MUNICH**	**DUSSEL**	**CPTAC**
**NLCS**	0.93	0.92	0.93	0.93	0.93	0.95	0.88	0.78	0.92	0.72	0.89	0.85	0.83	0.89
**DACHS**	0.91	0.96	0.91	0.92	0.93	0.92	0.86	0.76	0.91	0.74	0.87	0.83	0.83	0.87
**TRANSCOT**	0.91	0.91	0.93	0.93	0.92	0.95	0.87	0.76	0.93	0.75	0.89	0.86	0.80	0.85
**QUASAR**	0.93	0.92	0.93	0.96	0.93	0.95	0.89	0.76	0.93	0.72	0.88	0.88	0.81	0.91
**MCO**	0.92	0.91	0.92	0.93	0.94	0.94	0.88	0.78	0.92	0.75	0.87	0.87	0.82	0.88
**YCR-BCIP**	0.90	0.87	0.91	0.91	0.92	0.95	0.86	0.76	0.91	0.76	0.87	0.86	0.82	0.92
**FOxTROT**	0.83	0.82	0.89	0.82	0.80	0.87	0.81	0.70	0.84	0.71	0.80	0.77	0.79	0.82
**MECC**	0.85	0.82	0.88	0.86	0.85	0.88	0.81	0.77	0.86	0.68	0.81	0.77	0.80	0.79
**Epi700**	0.90	0.90	0.92	0.93	0.93	0.94	0.87	0.77	0.95	0.72	0.87	0.88	0.80	0.88
**ERLANGEN**	0.80	0.81	0.86	0.78	0.80	0.85	0.77	0.66	0.82	0.76	0.76	0.80	0.77	0.68
**TCGA**	0.82	0.86	0.84	0.85	0.83	0.88	0.80	0.72	0.85	0.70	0.83	0.84	0.76	0.82
**MUNICH**	0.77	0.78	0.79	0.80	0.80	0.84	0.74	0.65	0.81	0.72	0.76	0.85	0.76	0.71
**DUSSEL**	0.72	0.75	0.83	0.75	0.71	0.81	0.74	0.65	0.74	0.70	0.69	0.67	0.71	0.69
**CPTAC**	0.74	0.74	0.78	0.74	0.71	0.81	0.72	0.67	0.77	0.70	0.69	0.67	0.71	0.73
